# Developing whole cell standards for the microbiome field

**DOI:** 10.1186/s40168-022-01313-z

**Published:** 2022-08-09

**Authors:** Chrysi Sergaki, Saba Anwar, Martin Fritzsche, Ryan Mate, Robert J. Francis, Kirsty MacLellan-Gibson, Alastair Logan, Gregory C. A. Amos

**Affiliations:** 1 Division of Bacteriology, National Institute for Biological Standards and Control, Potters Bar, Hertfordshire, EN6 3QG UK; 2 Division of Analytical and Biological Sciences, National Institute for Biological Standards and Control, Potters Bar, Hertfordshire, EN6 3QG UK

**Keywords:** Microbiome, Standardisation, Bioinformatics, Reference reagents, Standards, DNA extraction, Next-generation sequencing

## Abstract

**Background:**

Effective standardisation of the microbiome field is essential to facilitate global translational research and increase the reproducibility of microbiome studies. In this study, we describe the development and validation of a whole cell reference reagent specific to the gut microbiome by the UK National Institute for Biological Standards and Control. We also provide and test a two-step reporting framework to allow microbiome researchers to quickly and accurately validate choices of DNA extraction, sequencing, and bioinformatic pipelines.

**Results:**

Using 20 strains that are commonly found in the gut, we developed a whole cell reference reagent (WC-Gut RR) for the evaluation of the DNA extraction protocols commonly used in microbiome pipelines. DNA was first analysed using the physicochemical measures of yield, integrity, and purity, which demonstrated kits widely differed in the quality of the DNA they produced. Importantly, the combination of the WC-Gut RR and the three physicochemical measures allowed us to differentiate clearly between kit performance. We next assessed the ability of WC-Gut RR to evaluate kit performance in the reconstitution of accurate taxonomic profiles. We applied a four-measure framework consisting of Sensitivity, false-positive relative abundance (FPRA), Diversity, and Similarity as previously described for DNA reagents. Using the WC-Gut RR and these four measures, we could reliably identify the DNA extraction kits’ biases when using with both 16S rRNA sequencing and shotgun sequencing. Moreover, when combining this with complementary DNA standards, we could estimate the relative bias contributions of DNA extraction kits vs bioinformatic analysis. Finally, we assessed WC-Gut RR alongside other commercially available reagents. The analysis here clearly demonstrates that reagents of lower complexity, not composed of anaerobic and hard-to-lyse strains from the gut, can artificially inflate the performance of microbiome DNA extraction kits and bioinformatic pipelines.

**Conclusions:**

We produced a complex whole cell reagent that is specific for the gut microbiome and can be used to evaluate and benchmark DNA extractions in microbiome studies. Used alongside a DNA standard, the NIBSC DNA-Gut-Mix RR helps estimating where biases occur in microbiome pipelines. In the future, we aim to establish minimum thresholds for data quality through an interlaboratory collaborative study.

Video Abstract

**Supplementary Information:**

The online version contains supplementary material available at 10.1186/s40168-022-01313-z.

## Background

The expansion of the microbiome field over the last decade has shed new light on the role of microbes in human health and disease. Methods in microbiome research are constantly evolving, and currently, there are multiple different approaches to study the microbiome [1–3]. The most common approach to studying the microbiome is to extract total community DNA from a sample, perform next-generation sequencing (NGS) on this DNA using either a 16S rRNA or shotgun metagenomic approach, and analyse this data using bioinformatic tools to reconstruct the microbial composition in the original sample. Notably, various methods can be used at each of these steps, most of which give different outputs. This technical variability causes considerable problems in the reproducibility of studies and can lead to contradicting results from conceptually similar studies [3–9].

Consortia such as the International Human Microbiome Standards (IHMS) group and the Microbiome Quality Control (MBQC) project have identified that variation between DNA extraction protocols is responsible for substantial variability between microbiome profiling strategies [5, 6]. Multiple comparative studies have described the impact of DNA extraction kits on microbiome profiling, leading to completely different conclusions for the same samples [10–14]. Variation between DNA extraction protocols can be attributed to differing abilities of kits to lyse gram-positive and gram-negative bacteria, contaminants in the reagents or equipment used, and interlaboratory and intra-laboratory operator differences even when using automated equipment [8, 14–18].

Considering there are over 130 active phase II clinical trials and 35 active phase III clinical trials investigating the microbiome or microbiome therapeutics such as faecal microbiota transplant (FMT), it is of great concern that microbiome data cannot be reliably reproduced or compared across studies. Standardisation of the microbiome field is urgently needed to harmonise results across methodologies and studies to allow for effective translation of research into the clinic. Some efforts have been made to standardise the field, with several authors advocating the use of positive and negative controls for sampling, DNA extractions, and NGS and bioinformatics, as well as the use of universal DNA extraction protocols across studies to reduce bias [4, 7, 8, 19–22]. However, to date, we are unaware of any of these recommendations leading to accredited or certified reference reagents, which are critical if there is to be effective standardisation of the microbiome space. Previously, we set out the strategy of the National Institute for Biological Standards and Control for effective standardisation of the microbiome field using reference reagents for control of sampling, DNA extractions, and NGS and bioinformatic workflows [3]. Physical reference reagents are the bedrock of standardisation and, if used appropriately, can negate the need for prescribed methodologies and encourage innovation by guaranteeing the quality of data using thresholds or minimum quality criteria (MQC).

In our recent work, we described the production and validation of a DNA reference reagent (NIBSC DNA-Gut-Mix RR) to standardise NGS and bioinformatic approaches for gut microbiome research [3]. This was based on a mock community of 20 strains specific for the gut microbiome with minimum quality criteria measured as Sensitivity, false-positive relative abundance, Diversity, and Similarity. Here, we describe the production and validation of a counterpart reagent to be used to standardise DNA extractions for study of the gut microbiome, herein after termed whole cell reference reagent (WC-Gut RR). We evaluated eight commercial DNA extraction kits most commonly used in human gut microbiome research using the WC-Gut RR and expanded the reporting system to consist of additional minimum quality criteria to ensure the physical quality of DNA. The WC-Gut RR can be used in combination with the NIBSC DNA-Gut-Mix RR to identify sources of bias and to measure levels of accuracy for all microbiome pipeline steps post sample collection. If widely adopted and used with appropriate minimum quality criteria, NIBSC WC-Gut RR will allow for reproducibility of work and enable comparisons across microbiome studies.

## Methods

### Growth and fixation of strains for reference reagents

Twenty strains representing common microbiota of the gut and used previously in DNA-based reagents NIBSC DNA-Gut-Mix RR were obtained from the Leibniz Institute DSMZ — German Collection of Microorganisms and Cell Cultures GmbH (DSMZ, Germany) (Table 1). Strains were cultured as recommended by DSMZ. All strains were checked to ensure they were in vegetative growth stage to allow for reproducibility and consistency across reagents. In the case of *Clostridium butyricum*, this required cells to be incubated overnight in a germination medium (100 mmol/L L-cysteine — HCL, 50 mmol/L NaHCO3, 10 mmol/L glucose) (Sigma-Aldrich, UK). Individual bacterial cultures were centrifuged at 3000 × *g* for 10 min once they reached their stationary phase, and the supernatant was replaced by 95% acetone (Sigma-Aldrich, UK) with 5% phosphate-buffered saline (PBS), containing 10 mM Tris. Bacteria were incubated in 95% acetone for 10 min and then centrifuged at 3000 × *g* for 3 min. Fixed bacteria were washed in PBS (10mM Tris) and stored in 1% trehalose (Sigma-Aldrich, UK) in PBS (10mM Tris) at 4 °C. Fixation was confirmed by viability counts using agar plates inoculated with 10^7^ bacteria. An absence of growth was used as an initial measure of successful fixation. Confirmation that the bacterial cells were not lysed and that the nucleic acids remained within the cell after the acetone fixation was provided by light and electron microscopy analysis.

**Table 1:**
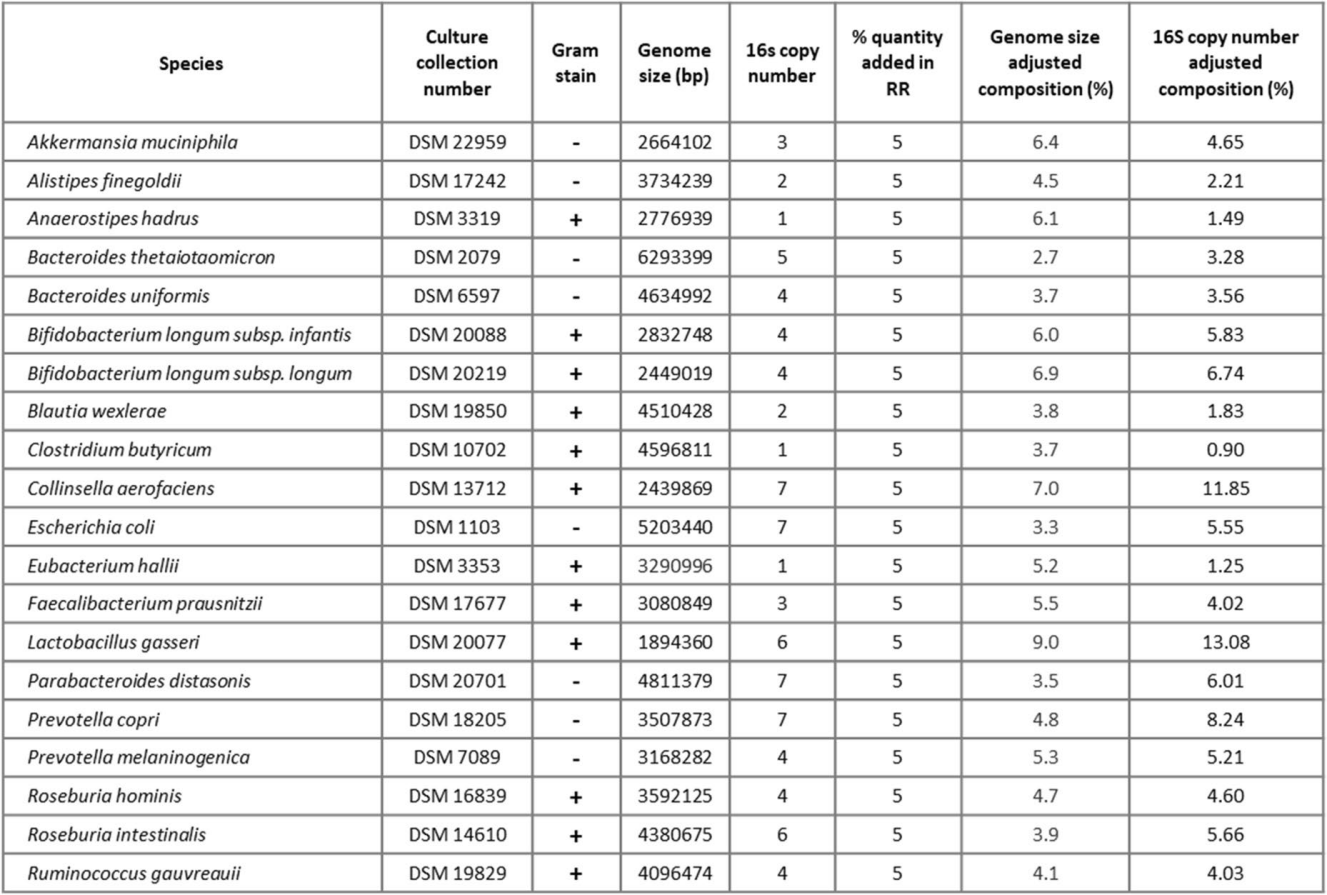
Strains used in the WC-Gut RR, their DSMZ culture collection number, the gram stain (+ for positive and − for negative), the genome size (bp), the 16S rRNA copy number, the amount of ng added in the reagent (serving as ground truth for the WC-Gut RR), the genome size adjusted composition (%) (serving as ground truth for the DNA-Gut-Mix RR), and the relative composition when adjusted for 16S copy number (%). 16S rRNA copy number is based on analysis of genome sequences through IMG/M

### Validity checks for cell wall integrity

For light microscopy, samples of the fixed bacterial cell mix were stained with acridine orange (AO) (Sigma-Aldrich, UK) at 10 μg/ml for 10 min to stain nucleic acids. Samples were imaged using 63×/1.2 NA H2O immersion lens on the Leica SP8X confocal laser scanning microscope (CLSM) (Leica Microsystems, UK). Images presented are maximum intensity projections of confocal Z stacks, with DNA imaged at excitation 488 nm, and emission 500–550 nm and RNA imaged at excitation 488 nm and emission at 600–650 nm.

For electron microscopy, samples of the fixed cell mix pre and post lyophilisation were high-pressure frozen in a Leica HMP 010 high-pressure freezer (Leica, Austria) with 20% HMW dextran average *M*_r_ ~70,000 (Sigma-Aldrich, UK) acting as a cryoprotectant. These samples were freeze-substituted into HM20 resin (Polysciences, Inc., PA) containing 2% uranyl acetate. Sections of 100 nm thickness were cut using an UC6 (Leica Microsystems Ltd., UK) fitted with a diamond knife (Diatome, Switzerland) and mounted on carbon-coated copper grids (Agar Scientific, UK). Sections were imaged in a JEOL JEM 2100 electron microscopes (JEOL (UK) Ltd., UK) running at 200 kv at magnifications ranging from 400× to 25,000×. Images were captured on a Gatan US4000 CCD camera running Digital Micrograph software (Gatan Inc., CA).

### Purity validation and enumeration of individual strains

Following microscopy, to confirm strain identity and perform additional checks of purity, DNA was extracted using the Qiagen DNeasy PowerSoil Kit (Qiagen, UK). PCR was performed using the universal 27F and 1492R primers and the Platinum Taq DNA polymerase (Thermo Fisher Scientific, UK). PCR products were purified using the QIAquick PCR Purification Kit (Qiagen, UK) and sent for Sanger sequencing at Source Biosciences, Cambridge. Resulting sequences were BLAST searched against the NCBI nr/nt database for strain identification. DNA was tested for purity using shotgun sequencing. Libraries for shotgun sequencing were constructed using the Nextera DNA Flex Library Prep Kit (Illumina, 20018705) and sequenced paired-end with 150 bp read length on a NextSeq 500 platform (Illumina), using the High Output kit (Illumina, 20024908). Shotgun data was analysed as previously described [3]. In brief, we used FastQC (v0.11.9) [23] for data quality assessment. BBDuk (v37.62) [24] was used for filtering of the sequences, with quality trimming at *Q* = 25 and minimal length filtering of trimmed reads at 100 bp. Following quality control, shotgun sequencing data was analysed using MetaPhlAn3 (v3.0) [25].

To combine the 20 strains in equal ratios, the number of fixed cells for each bacterial strain was enumerated using flow cytometry, the gold standard method for such purposes [26–32]. Strains were quantified using the Bacteria Counting Kit for flow cytometry (Invitrogen, UK) in accordance with the manufacturer’s instructions. In brief, the flow cytometer FACSCanto II (BD Bioscience, UK) and BD FACSDiva software v8 (BD Bioscience, UK) were used. Excitation was set to 324V for FSC, 319V for SSC, and 352V for FITC, and the threshold for FITC was set to 200. Three replicates were quantified for each sample containing the dye and the beads, as well as one sample with no dye. A total of 10,000 events or 30 s were recorded for each sample. Samples were analysed using FlowJo v10 software. The signal from the buffer and from the unstained bacteria was eliminated from the measurements. In order to determine the cell number per ml, the samples were analysed as recommended by the Bacteria Counting Kit, for flow cytometry (Invitrogen, UK).

### Lyophilisation of strains mix to create WC-Gut RR

Based on the number calculated using flow cytometry, the 20 strains were combined in equal ratios to a final concentration of 2 × 10^9^ cells/ml in 1% trehalose in PBS (10 mM Tris) and freeze-dried using a 2-day freeze-drying cycle. Trehalose is known as an effective nucleic acid preservative and cryoprotectant [33]. The resulting lyophilised strain mix constitutes the WC-Gut RR.

### Assessment of multiple DNA extraction kits

The WC-Gut RR was reconstituted using 500 μl PBS resulting in a concentration of 4 × 10^9^ cells/ml. Five replicates of WC-Gut RR were extracted using eight DNA extraction kits (Table 2). DNA extractions were performed according to manufacturer’s recommendations. The pre-lyophilised mix was also extracted with the same kits in order to assess the effect of lyophilisation on the DNA extractions. DNA extractions were also performed on commercially available materials, ZymoBIOMICS™ Microbial Community Standards (ZYMO D6300, lot ZRC187326), 20 Strain Even Mix Whole Cell Material (ATCC® MSA-2002™, lot 70003365), 10 Strain Even Mix Whole Cell Material (ATCC® MSA-2003™, lot 70003364), and Gut Microbiome Whole Cell Mix (ATCC® MSA-2006™, lot 70019370). The reagents were reconstituted and used as recommended by the manufacturers.

**Table 2:**
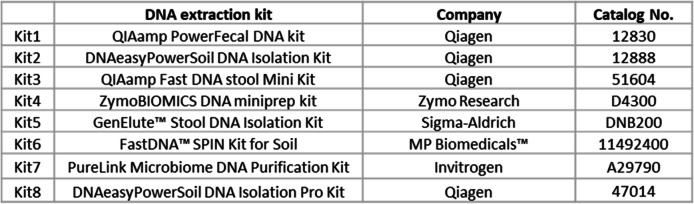
DNA extraction kits used in the study

### Physicochemical characterisation of DNA

DNA yield was assessed using Qubit fluorometric quantification for high sensitivity double-strand DNA quantification (Thermo Fisher Scientific, UK) following the manufacturer’s instructions. The DNA Integrity Number (DIN) was measured using Agilent 2200 TapeStation and Genomic DNA TapeStation reagents (Agilent, UK). The purity of the DNA was assessed using spectrometry via SpectraMax QuickDrop Micro-Volume Spectrophotometer (Molecular Devices, UK), recording the 260/280 nm ratio. The statistical tests used to identify any significant differences between kits were pairwise *t*-test and ANOVA followed by post hoc Tukey with FDR-adjusted *p*-value.

### Next-generation sequencing

Extracted DNA was sequenced using shotgun sequencing to evaluate microbiome composition. Libraries for shotgun sequencing were constructed using the Nextera DNA Flex Library Prep Kit (Illumina, USA) and sequenced paired end with 150 bp read length on a NextSeq 500 platform (Illumina, USA), using the High Output kit (Illumina, USA). DNA was also subject to 16S rRNA sequencing. Libraries for 16S rRNA were constructed by amplifying the V4 region of 16S rRNA amplicon using the primers 515F(Parada)/806R(Apprill) [34, 35], as indicated by the Earth Microbiome Project (EMP [36];) with PCR performed using the Platinum Taq DNA Polymerase (Thermo Fisher Scientific, UK) for 35 cycles with annealing temperature of 50 °C and extension time of 90 s. Illumina sequencing adapters and dual-index barcodes were added to the purified 16S rRNA PCR amplicons, followed by a clean-up and size selection according to Illumina protocol 15044223 (Rev. B). Libraries were sequenced using an Illumina MiSeq with 250 bp paired-end reads.

### Bioinformatic analysis of shotgun sequencing data

Shotgun data was analysed as previously described [3]. In brief, we used FastQC (v0.11.9) [23] to make initial judgements on data quality. BBDuk (v37.62) [24] was used for quality control of the sequences, with quality trimming at *Q* = 25 and minimal length filtering of trimmed reads at 100 bp. Following quality control, shotgun sequencing data was analysed using either MetaPhlAn3 (v3.0) [25], Kaiju (v1.7.2 ) [37], Centrifuge (v1.0.3 ) [38], Bracken (v2.5) [39], or Kraken (v1.1.1) [40], in line with the developer’s recommendations in their tool’s tutorials (i.e. default) and using the recommended database for each tool (see Additional file 5, the standard Kraken (v1) database was used for Kraken and Bracken). For MetaPhlAn3 and Centrifuge, the forward and reverse reads were combined into a single file before being processed. Outputs were used to generate species abundance tables using excel and R [41]. More details can be found in the supplementary methods, Additional file 5.

### Bioinformatic analysis of 16S rRNA sequencing data

16S rRNA sequencing data was analysed as previously described [3]. Data was analysed using QIIME2 (v2020.2) with Deblur [42]. In brief, primers and adapters were removed with the q2-cutadapt plugin, and paired-end reads were joined using the q2-vsearch plugin. Sequences were then quality controlled using the q2-quality-filter plugin followed by the q2-deblur plugin for denoising. Following this, the q2-feature-classifier (sklearn) was used to assign taxonomy to representative sequences against the Silva database (132 release). Sequences were further filtered using the q2-feature-table plugin to ensure that all features which were less than 0.005% abundant for each replicate are removed [43]. The q2-taxa plugin was used to generate taxa bar plots which were used to extract relative genera abundances within each sample. A comprehensive overview of the methods can be found in Additional file 5.

### Calculation of the four reporting measures

Using the abundance tables generated through bioinformatic analysis of the shotgun and 16S rRNA sequencing data, the measures of Sensitivity, false-positive relative abundance (FPRA), Diversity, and Similarity were calculated in accordance to Amos et al. (2020). The Sensitivity, FPRA, and Diversity were analysed in Excel using the following equations:

$$Sensitivity=\frac{Number\ of\ correctly\ identified\ species}{Total\ number\ of\ species\ in\ reagent} \times 100$$

$$False- positive\ relative\ abundance=\frac{Abundance\ of\ all\ false- positive\ species}{Total\ abundance\ of\ all\ species}\times 100$$

$$Diversity= Total\ number\ of\ all\ observed\ species\ \left( true\ positive+ false\ positive\right)$$

Similarity was calculated using the vegdist function in the R (v3.60) vegan package (v2.5.7).

$${Similarity}_{ij}=\frac{2{C}_{ij}}{S_i+{S}_j}$$

     where i is the known species profile of the reagent, j is the predicted species profile of the reagent from the tested analytical pipeline, and C_ij_ is the sum of only the lesser abundance for each species found in both the known species profile of the reagent and the predicted species profile of the reagent from the tested analytical pipeline.

## Results

### Development of NIBSC Gut Whole Cell Reference Reagent (WC-Gut RR)

Bacterial strains included in the NIBSC Gut Microbiome Whole Cell Reference Reagents (WC-Gut RRs) were the same 20 strains used for the NIBSC DNA-Gut-Mix RR [3] to facilitate complementary usage. Strains encompass 5 phyla, 13 families, 16 genera, and 19 species ensuring a wide range of cell wall types representing what would commonly occur in the gut. They also represent a taxonomic identification challenge for downstream sequencing and bioinformatics, similar to challenges during gut microbiome analysis. Twelve strains are gram positive, and eight strains are gram negative.

**Fig. 1:**
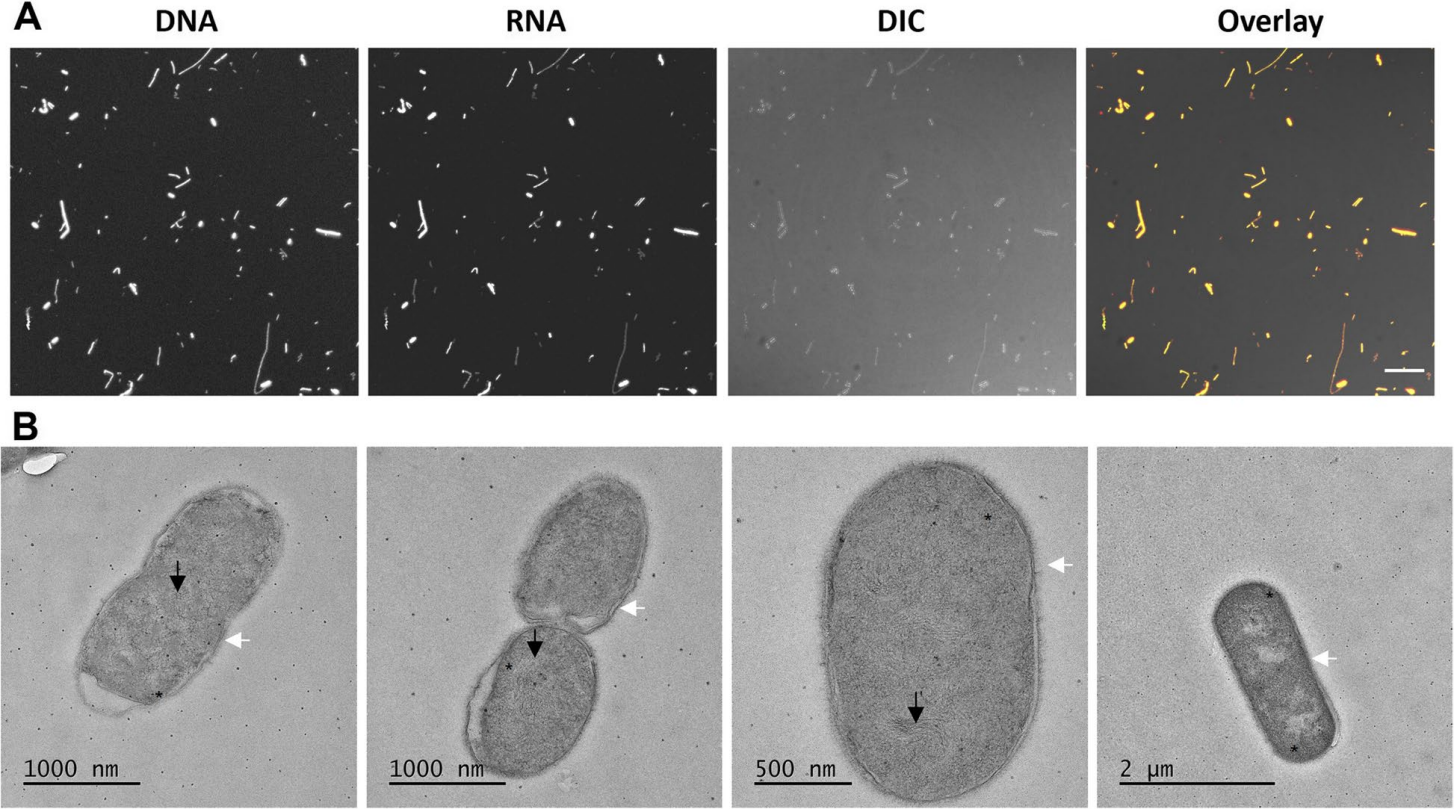
Microscopy images indicating that cells are intact with the DNA within the cells after fixation. **A** Confocal microscopy images of acetone fixed bacteria stained with acridine orange (AO), setting adjusted to allow observation of DNA, RNA, differential interference contrast (DIC), and an overlay of all three. Images presented are maximum intensity projections of confocal Z stacks. **B** EM images of bacterial cells, black arrows indicate the presence of granular nucleoplasm with fibrillar whorls inside the cell membranes, indicating that DNA is preserved within the cells post fixation. White arrows indicate the presence of cell walls

For stability and distribution purposes, bacteria were grown in liquid culture and fixed with 95% acetone. Acetone was chosen as a fixative agent as it has a demonstrated ability to fix the cell and preserve the DNA [44]. To ensure that acetone fixation did not change the extraction efficiency of cells, we performed DNA extractions on fixed and unfixed pure cultures. No significant difference was observed in DNA extraction efficiency between unfixed and fixed bacteria cells (pairwise *t*-test, FDR-adjusted *p*-value, see Supplementary Table 1, Additional file 1). The absence of bacterial growth for all final reagents was confirmed through culture-based viability testing. Furthermore, confocal and electron microscopy demonstrated that bacterial cell maintained their cell form, with both cellular DNA and RNA identified within the cells, observed as fine fibrillar structures (Fig. 1). The formation of these fine fibrillar structures is caused by the sample preparation method of chemical fixation combined with freeze substitution, which results in the aggregation of DNA within the nucleoplasm [45, 46]. Freeze-drying on the reagents did not influence the consistency of the reagent (i.e. the microbiome composition) nor the DNA extraction efficiency of the kits (see Supplementary Table 2, Supplementary Fig. 1 and 2, Additional files 1, 2, and 3, respectively).

### Analysis of the variability in physicochemical characteristics of DNA across different extraction methods using Gut-WC RR

WC-Gut RR aims to standardise the DNA extraction step in gut microbiome analytical pipelines and highlight where biases occur. Therefore, we required a suitable reporting framework that scientists can use to establish that their methodologies and pipelines are fit for purpose. We developed a two-step framework that firstly assessed physicochemical data to ensure DNA is fit for the purpose of downstream sequencing and secondly assessed the taxonomic composition using a four-measure reporting system as previously described [3]. By employing this framework, users can save time and costs by first assessing the DNA quality prior to sequencing to determine composition.

For reporting of DNA physicochemical quality and quantity, we proposed three measures that assess total DNA yield, DNA integrity, and purity as measured by contamination of protein. Total DNA yield was measured by fluorimetry using a Qubit. DNA integrity was measured by the DNA Integrity Number (DIN) as measured by Agilent TapeStation since this gives a clear numerical value of DNA degradation based on electrophoresis. Finally, purity was assessed using spectrophotometry, specifically absorbance at 260 nm and 280 nm, with a ratio of 260/280 used to indicate purity. This was used on the basis that a 260/280 nm ratio of ~1.8 is widely accepted as a measure of pure DNA [47].

**Fig. 2:**
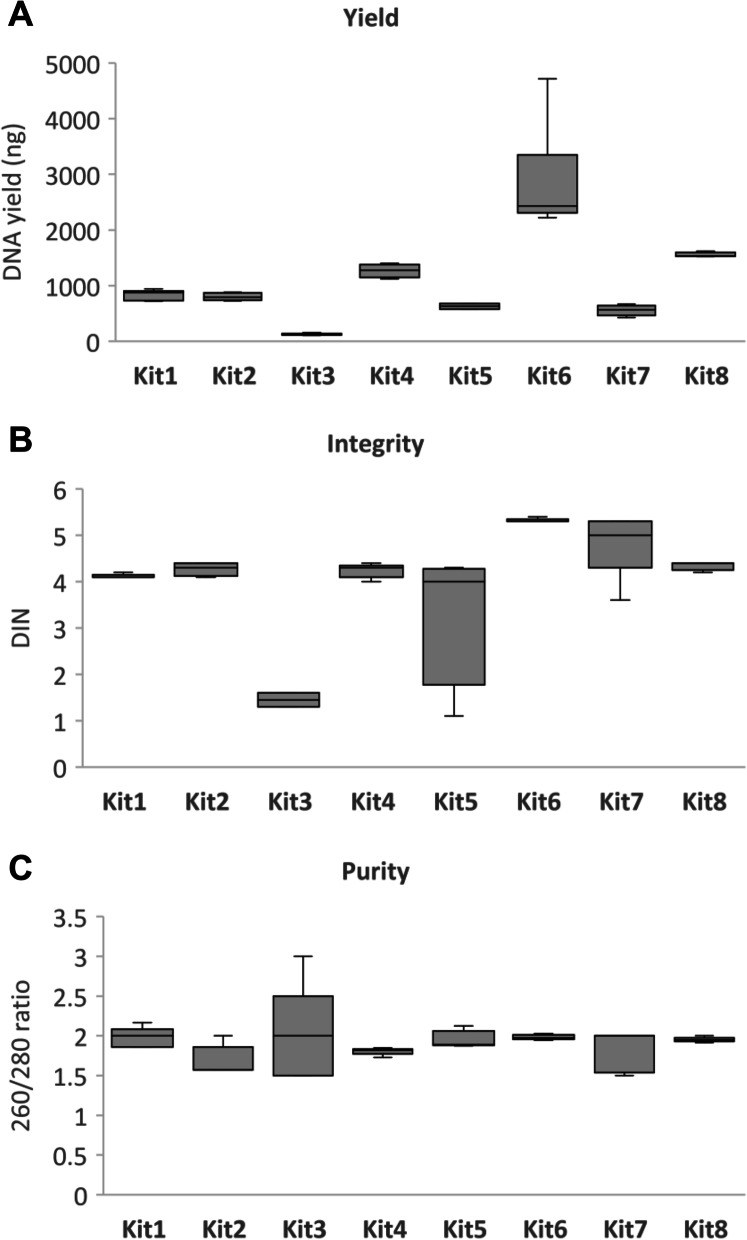
Physicochemical measures of DNA extracted from the NIBSC WC-Gut RR using eight commercial DNA extraction kits, based on the average of five replicates. **A** DNA yield measured as ng of DNA. **B** DNA integrity measured using the DNA Integrity Number (DIN). **C** DNA purity as measured by absorbance ratio at 260 nm/280 nm. The top and bottom of the box represent the third quartile and first quartile data values, while the horizontal top and bottom line at the end of the whiskers show the maximum and minimum data value respectively

We assessed the suitability of WC-Gut RRs and the two-step reporting framework using eight DNA extraction kits commonly used in microbiome studies. Chosen kits are produced by various manufactures and use different strategies to extract nucleic acids from bacteria (see Supplementary Table 3, Additional file 1). Significant differences were observed in the mean average DNA yield depending on the kit (Tukey HSD following ANOVA, FDR-adjusted *p*-value < 0.05, except for kits 1, 2, and 5, as well as Kit 5 and Kit 7 that had similar yield) with a range from 2462 ng (Kit 6) to 125 ng (Kit 3) (Fig. 2). Six of the eight kits extracted DNA of similar integrity with DIN scores ranging from 4.12 to 5.32. However, Kit 5 had a notably low DIN score of 3.35, as a result of the very low DNA concentration. Similarly, due to the consistent poor yield of Kit 3, no integrity analysis was possible, as the TapeStation’s limit of detection is 1 ng/μl. Seven of the eight kits had a consistent mean average of > 1.8 260/280 absorbance ratio with Kit 2 slightly lower at 1.69. Collectively, these results demonstrate that Gut-WC RRs can help to compare the yield, integrity, and purity of DNA extracted using different commercial DNA extraction kits. The exact threshold for what users should achieve is dependent on the downstream sequencing technology to be used, with long-read technologies favouring intact and pure DNA. A potential limitation is that extractions from stool will have inhibitors and enzymes causing degradation. However, by using this reagent, users can consistently gauge how well a DNA extraction has worked across experiments and studies and be confident that inadequate readings for the three measures on a whole cell reagent will almost certainly reflect extraction failure for their samples. Notably, all DNA extractions using the WC-Gut RR were shown to be reproducible, with average coefficient of variation (CV) values being below 100% for all measures (see Supplementary Table 4, Additional file 1).

### Analysis of variability in community composition of DNA across different extraction methods using WC-Gut RRs

We next assessed whether the WC-Gut RRs could be used to evaluate how well different DNA extraction kits preserve the composition of the community in the reagent. To do this, we used a four-measure reporting system, as previously developed for evaluating next-generation sequencing and bioinformatic pipelines for NIBSC DNA-Gut-Mix RR, a complementary DNA reagent [3]. This allows for a comprehensive analysis of how well a methodology detects strains, prevents false positives, and accurately reconstitutes the community composition of the target sample. Measures calculate the number of strains detected (Diversity), the Bray-Curtis Similarity between the community composition given by the pipeline and the actual composition of the reagent (Similarity), the number of correct strains detected (Sensitivity), and the relative abundance of false-positive identifications in the final dataset (false-positive relative abundance, FPRA). Further details of the rationale behind these measures are discussed exhaustively in previous work [3].

**Fig. 3:**
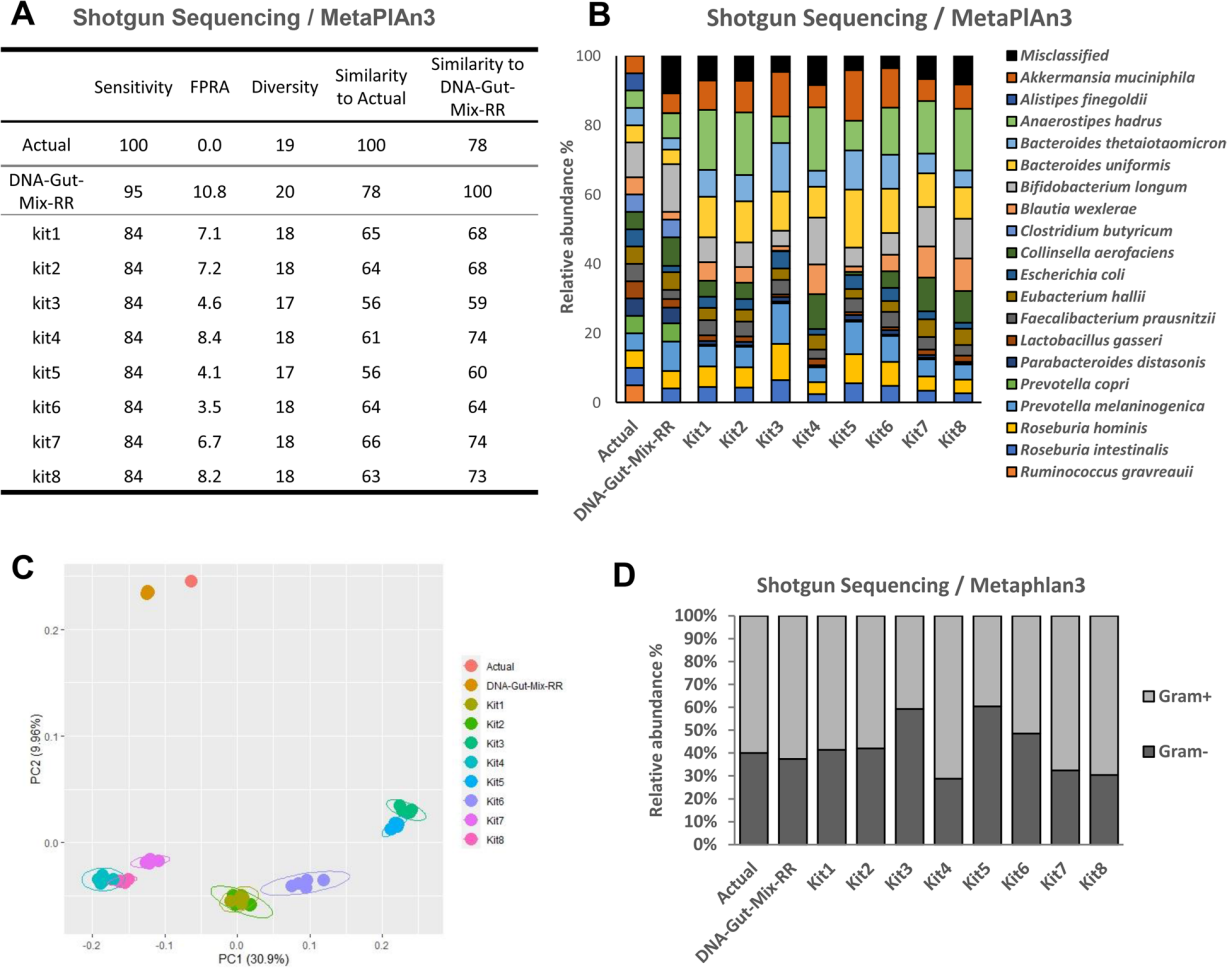
Comparison of DNA extraction kits and their extraction biases evaluated using shotgun sequencing and MetaPhlAn3. Samples analysed are the Actual (ground truth), the NIBSC DNA-Gut-Mix RR, and DNA extracted from the WC-Gut RR using Kit1, Kit2, Kit3, Kit4, Kit5, Kit6, Kit7, and Kit8, based on the average of five replicates. **A** Values of each sample using the four-measure reporting system consisting of Sensitivity, False-Positive Relative Abundance (FPRA), Diversity, Similarity to Actual composition, and Similarity to the NIBSC DNA-Gut-Mix RR. **B** Relative abundance of (%) each species in each sample. **C** β-diversity analysis using Bray-Curtis dissimilarity measure visualised by a principal coordinate analysis. **D** Relative abundance (%) of gram-positive and gram-negative bacteria recovered using each kit

Previous work using NIBSC DNA-Gut-Mix RR demonstrated that MetaPhlAn3 had the best analytical performance according to the four-measure reporting system [3]. We therefore initially analysed taxonomic profiles of extracted DNA using shotgun sequencing paired with MetaPhlAn3 and demonstrated significant differences across the various DNA extraction kits used (Fig. 3). Using the four-measure reporting system, we compared the MetaPhlAn3 results to the samples’ actual compositions (Fig. 3A). Differences introduced by the kits were observed for FPRA, Diversity, and Similarity, whereas Sensitivity was constant at 84% (16/19 species) for all kits. While the species *Ruminococcus gauvreauii* was also absent from the DNA-Gut-Mix RR, i.e. it was absent due to bioinformatics bias, the species *Clostridium butyricum* and *Alistipes finegoldii* were absent only in the WC-Gut RR. That indicates bias during the DNA extraction process for these two strains since they were present in the DNA-Gut-Mix RR. *Clostridium butyricum* and *Alistipes finegoldii* were present in the 16S amplicon sequencing results in small amounts (0–0.01%), indicating only traces of DNA were extracted and could only be identified through the PCR amplification of the 16S rRNA region but not via the shotgun sequencing methodology. FPRA ranged from 3.5 (Kit 6) to 8.2% (Kit 8). Diversity was 18 for kits 1, 2, 4, 6, 7, and 8 and 17 for Kit 3 and Kit 5, both of which had the lowest DNA concentration as assessed through physicochemical analysis. We next assessed Similarity, which was significantly different between kits (PERMANOVA, *p*-value < 0.05, see Supplementary Table 6, Additional file 1), with kits 1,2,6, 7, and 8 ranging between 63 and 66% similarity to the ground truth. Given equivalent levels of Sensitivity, Diversity and Similarity, these five kits performed best, with Kit 6 leading to lower levels of FPRA but decreased levels of Similarity relative to kits 1 and 7. Collectively, these results demonstrate how the NIBSC WC-Gut RR can help in determining the ability of different DNA extraction kits to accurately represent microbiome composition.

Even with the highest performing DNA extraction kit according to the Similarity measure, a 34% reduction in similarity to the known ground truth was observed. To better understand the reasons for this, we investigated the results of the complementary DNA reagent, NIBSC DNA-Gut-Mix RR, which was sequenced in parallel. Previous studies have suggested that DNA extraction kits introduce the most bias into studies [6]. However, these studies have not separated the bioinformatic tool from the DNA extraction kit. By combining the results of DNA-Gut-Mix RR with WC-Gut RR, we clearly dereplicate that 22% loss of similarity from the known composition is introduced by bioinformatic tools. Using the DNA reagents, shotgun sequencing paired with MetaPhlAn3 was only capable of achieving 78% similarity to the known ground truth. However, as the similarity of the composition of DNA extracted from the WC-Gut RR is at best 74% similar to the NIBSC DNA-Gut-Mix RR, it is likely that even with a perfect sequencing and bioinformatic pipeline, the best results that could be achieved would be 68–74% Similarity to the ground truth when using DNA extraction kits 1,2,4,7, and 8.

In order to understand the differences between the DNA extraction kits, we performed a more detailed analysis of the changes in individual species (Fig 3B). Most of the strains were significantly different in Relative Abundance in comparison with the DNA-Gut-Mix RR (Kruskal-Wallis, FDR *p*-value < 0.05, see Supplementary Table 5, Additional file 1). Significant differences were also observed in β-diversity (Fig. 3C), with the microbiome resulted from the extraction of the WC-Gut RR having significantly different β-diversity in comparison with the DNA-Gut-Mix RR, confirming the results observed using measures of Similarity. When grouping strains are based on their gram stain (Fig. 3D), analysis demonstrated significant differences (Tukey HSD following ANOVA, FDR-adjusted *p*-value < 0.001, see Supplementary Table 7, Additional file 1) in the ability of the different kits to extract DNA from gram-positive and gram-negative bacteria, supporting results of studies prior [17, 18]. Two pairs of kits, Kit 1 and Kit 2 and Kit 4 and Kit 8, had a similar ability in extracting DNA from gram-positive and gram-negative bacteria. Kits 3 and 5, which had the worst performance in the physicochemical measures and the four measures of taxonomic composition, had a poor ability to extract DNA from gram-positive bacteria (Fig. 3D and Supplementary Table 7, Additional file 1).

Previous studies have demonstrated a reagent-derived contamination present with some extraction kits [15, 23, 48]. Using negative controls, MetaPhlAn3 did not map any sequences for the negative control samples for any of the DNA extraction kits. Despite this, we would still recommend all studies incorporate negative controls into their extractions.

Our past work with NIBSC DNA-Gut-Mix RR demonstrated MetaPhlAn3 was the most specific bioinformatic tool (by the measure of FPRA) and had the highest similarity of microbiome community to the actual composition when using DNA reference reagents [3]. As a follow-up step, we tested whether individual DNA extraction kits may perform better with other bioinformatic pipelines, perhaps due to biases in pipelines correcting or masking biases introduced by different DNA extraction kits. We therefore compared different combinations of DNA extraction kits with different bioinformatic tools. Tools used included MetaPhlAn3, Centrifuge, Kaiju, Bracken, and Kraken (see Supplementary Fig. 3, Additional file 4). Using DNA extracted from the WC-Gut RR, results matched those previously described, with Sensitivity being reduced when using Centrifuge, Kraken, and Bracken, but increased when using Kaiju, in comparison with data produced by MetaPhlAn3. While the FPRA values varied between the different bioinformatics tools, the Diversity values where very different for the actual Diversity, with Centrifuge pipeline identifying 46–57 species in the reagent, Kaiju identifying 167–207 species, Kraken 68–84, and Bracken 72–95 species, in comparison with the 17–20 species identified using MetaPhlAn3 and the 19 species expected based on the actual composition of the sample. As previously shown by Amos et al. (2020), Centrifuge, Bracken, and Kraken have the lowest Similarity to the actual composition (Similarity < 60%, see Supplementary Fig. 3 A, C, and D, Additional file 4). MetaPhlAn3 and Kaiju demonstrated similarities to the actual composition of > 60%, with kits 1, 2, 6, and 7, all being between 64 and 66%. This suggests that kits 1, 2, 6, and 7 combined with MetaPhlAn3 give the most accurate analysis of the composition of a target sample, among the DNA extraction kits and bioinformatics tools tested. The observed differences between the bioinformatics tools may also be attributed to the design of the tools (e.g. MetaPhlAn3 being more conservative) and whether they report relative sequence abundance or relative taxonomic abundance [24].

### Compatibility of WC-Gut RRs with 16S rRNA sequencing

**Fig. 4:**
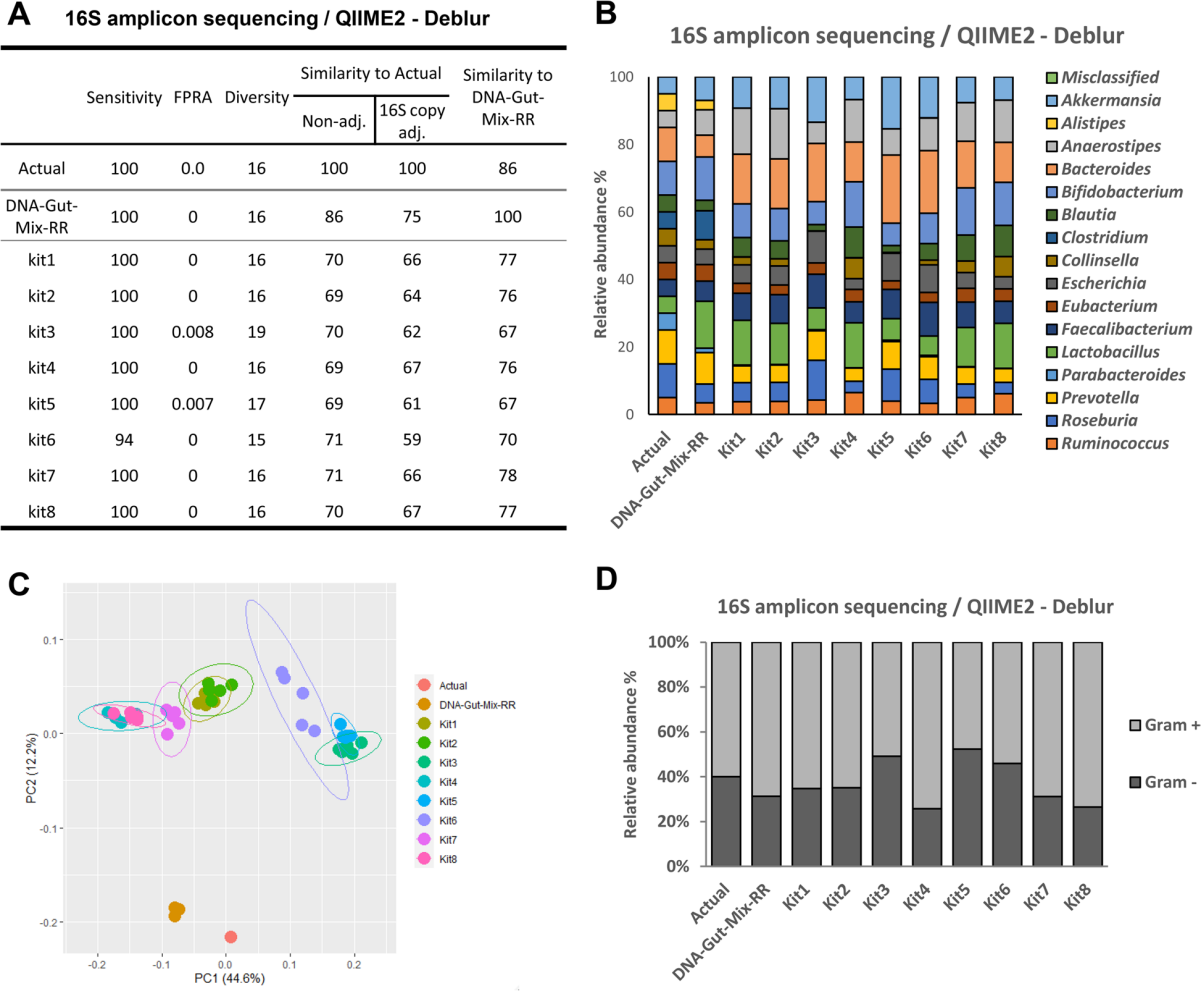
Comparison of DNA extraction kits and their extraction biases evaluated using 16S rRNA amplicon sequencing. Sequencing data were analysed with Deblur through QIIME2. Samples analysed are the actual (ground truth) with and without adjustment for 16S rRNA copy number, the NIBSC DNA-Gut-Mix RR, and DNA extracted from the WC-Gut RR using Kit1, Kit2, Kit3, Kit4, Kit5, Kit6, Kit7, and Kit8, based on the average of five replicates. **A** Values of each sample using the four-measure reporting system consisting of Sensitivity, False-Positive Relative Abundance (FPRA), Diversity, Similarity to Actual composition, and Similarity to the NIBSC DNA-Gut-Mix RR. **B** Relative abundance of (%) each genera in each sample. **C** β-diversity analysis using Bray-Curtis dissimilarity measure visualised by a principal coordinate analysis. **D** Relative abundance (%) of gram-positive and gram-negative bacteria recovered using each kit

Previous work using NIBSC DNA-Gut-Mix RR demonstrated the V4 region with analysis through QIIME2, and Deblur gave the highest levels of Sensitivity, FPRA, Diversity, and Similarity compared to the known composition. To ensure DNA extracted from WC-Gut RRs was compatible with 16S rRNA sequencing, we performed amplicon sequencing of the V4 region on the same DNAs extracted for shotgun sequencing with subsequent analysis with Deblur through the QIIME2 platform. Analysis at the genera level revealed differences across extraction kits in microbial diversity and composition (Fig. 4). The four taxonomic data measures (Sensitivity, FPRA, Diversity, and Similarity) were more consistent across kits than for shotgun sequencing data (Fig. 4A). This could be due to primer amplification bias offsetting extraction biases and thereby leading to a more even composition. Additionally, taxonomic identification is being set at a genera level giving a greater margin for error in taxonomic analysis by bioinformatic pipelines. Despite this, the reagents could clearly identify those kits that did not detect all genera and those reagents that led to elevated measures of Diversity. Specifically, Kit 6 had reduced Sensitivity with reference to all other kits, and all kits except Kits 3, 5, and 6 identified 16 as the number of genera present, the same as the actual composition. Similarity was consistently between 69 and 71% to the actual composition and 59–67 % when adjusting to 16S copy number, confirming previous work indicating that adjusting for 16S copy number does not improve the results [36]. Similarity between the results of the WC-Gut RR and the DNA-Gut-Mix RR ranged from 67 to 78 %. This suggests that part of the bias is due to library preparation, sequencing, and bioinformatics analysis. Improving the outcome from that part of the process, e.g. by using a bioinformatic pipeline of higher accuracy, may reveal the true level of bias introduced by the DNA extraction process, i.e. which microbes’ DNA was not efficiently extracted and therefore not appropriately represented in the sequencing results. Overall, when excluding the bioinformatics bias, by calculating the Similarity of the WC-Gut RR to the DNA-Gut-Mix RR, a 22–33% reduction in Similarity was observed. This is slightly improved Similarity in comparison with the shotgun sequencing data, most likely due to the different technologies and resolution at the genera level vs species level.

Similar to the shotgun sequencing results, analysis of the microbial composition (Fig. 4B) demonstrated nearly all of the genera were significantly different in relative abundance in comparison with the DNA-Gut-Mix RR (based on Kruskal-Wallis, FDR *p*-value < 0.05, see Supplementary Table 8, Additional file 1). Analysis of the β-diversity (Fig. 4C) also identified similar patterns to those found in the shotgun sequencing results. All the tested kits presented significantly different β-diversity in comparison with the DNA-Gut-Mix RR and to each other (based on PERMANOVA, *p*-value < 0.05, see Supplementary Table 9, Additional file 1). When the strains were grouped based on their gram stain (Fig. 4D), significant differences were identified in the ability of the kits to extract from gram-negative and gram-positive bacteria, relative to the DNA-Gut-Mix RR and each other (Tukey HSD following ANOVA, FDR-adjusted *p*-value, see Supplementary Table 10, Additional file 1). Post hoc testing demonstrated that two pairs of kits, Kit 1–Kit 2 and Kit 4–Kit 8, did not exhibit significant differences in the ability to extract DNA from gram-positive and gram-negative bacteria (Fig. 4D and Supplementary Table 10, Additional file 1).

In summary, these results demonstrate the ability of NIBSC WC-Gut RR to distinguish between the ability of DNA extraction kits using sequencing technologies but also highlight that using 16S rRNA sequencing and having a higher taxonomic resolution can reduce the appearance of biases in the dataset compared to shotgun sequencing.

### Impact of mock community composition on kit performance

Due to our findings that kits extracted gram-positive and gram-negative bacteria with different efficiencies, we explored how community composition influenced the performance of kits. For this purpose, we used the three highest performing DNA extraction kits, from different manufacturers, based on scores of Similarity to the Actual composition. Four different commercial whole cell reagents were extracted, shotgun sequenced, and analysed with MetaPhlAn3.

**Fig. 5:**
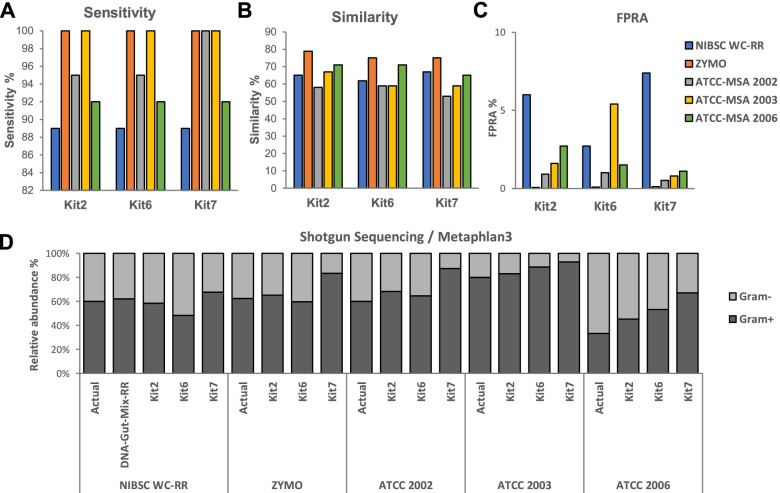
Comparison of selected DNA extraction kits when using mock communities of different microbial compositions. Kits selected were Kit 2, Kit 6, and Kit 7. Data is based on shotgun sequencing of DNA analysed using MetaPhlAn3. Mock communities used were NIBSC WC-Gut RR, ZymoBIOMICS Microbial Community Standards (ZYMO), 20 Strain Even Mix Whole Cell Material (ATCC-MSA 2002), and 10 Strain Even Mix Whole Cell Material (ATCC-MSA 2003). **A** Sensitivity. **B** Similarity in composition with the actual composition. **C** False-positive relative abundance (FPRA). **D** Relative abundance of gram-positive and gram-negative bacteria in the samples. The results are based on the average of three replicates

Kits demonstrated variable ability in extracting DNA from the five different communities as measured by Sensitivity, FPRA, and Similarity (Fig. 5). All kits had reduced Sensitivity for the NIBSC WC-Gut RR compared to other commercial reagents. Kits also had the highest FPRA for NIBSC WC-Gut RR compared to other reagents, with Similarity being broadly comparable across ATCC reagents and the NIBSC WC-Gut RR. This clearly demonstrates that the specific composition of the NIBSC WC-Gut RR poses a strong challenge to common microbiome pipelines. This highlights the importance of using reagents that are targeted and complex. Reagents that are of low complexity, well clinically characterised, and not representative of fastidious anaerobic bacteria that are observed in the gut will give users false confidence as to how well their pipelines are working. Kits significantly differed in their ability to extract DNA from gram-positive and gram-negative bacteria (Tukey HSD following ANOVA, FDR-adjusted *p*-value, see Supplementary Table 11, Additional file 1), depending on the strains used in the reagent (Fig. 5D, the reagent composition can be found in the Supplementary Table 12, Additional file 1). These results highlight the necessity of reagents that include strains with different lysis ability, and strains that are relevant to the microbiome of interest, in order to recapitulate more accurately the real bias introduced by the DNA extraction processes and downstream bioinformatics analysis.

## Discussion

The microbiome scientific community has highlighted the need for standardised pipelines, which are essential for the progression of the microbiome field and translating research into clinical practice [4, 6–8, 19, 49–51]. From the starting point of collecting a human microbiome sample, until the end point of having a profile of the microbial community, there are multiple steps that can each introduce bias into the results [6, 7, 18, 49, 52]. Multiple studies have indicated the bias introduced during the DNA extraction of microbiome samples leads to considerably different microbial profiles [10, 12–14]. It has been suggested that the DNA extraction process may have the largest effect on the outcome of metagenomic analysis [6]. In the effort to minimise the bias introduced during the DNA extraction process and to increase our confidence in data, we have developed the WC-Gut RR for the standardisation of the DNA extraction of microbiome samples. This reagent can act as a global working whole cell standard and is a candidate WHO International Reference Reagent for DNA extraction of microbiome samples. This is part of a broader strategy of NIBSC for effective standardisation of the microbiome field and complementary to the DNA reference reagents published previously [3].

Using eight commercial DNA extraction kits, from five different companies, with two different Next Generation Sequencing approaches, and six bioinformatics pipelines, we comprehensively demonstrate that WC-Gut RR allows for detecting biases in the DNA extraction step of microbiome pipelines. Importantly, WC-Gut RR could be used to detect significant differences in the ability of DNA extraction kits to extract from different strains, resulting in differing levels of Sensitivity, Similarity, FPRA, and Diversity. In the current study, we followed manufacturer’s instructions. This does not mean that the kits cannot be optimised for better usage, nor that there are not better kits in existence. The aim of this study was to demonstrate the utility of WC-Gut RR and the biases in microbiome pipelines that can occur, not prescribe a best standard procedure. In the views of the authors of this study, prescribing a fixed best practice protocol at an early stage in the field’s development can be problematic for innovation purposes. Instead, we advocate for an approach where people use physical reference reagents alongside quality criteria to validate a method as fit for purpose and allow for a high degree of comparability across studies. This will increase the confidence in the results and conclusions produced by microbiome studies and prevent the field from producing multiple incomparable datasets with unsubstantiated claims of ‘best methods’ that have not been independently validated.

The use of commercial whole cell reagents is increasing in the microbiome field. We therefore thought it important to assess how changing the composition of reagents can influence benchmarking studies. There were clear differences in the ability of the DNA extraction kits to extract DNA varied, depending on the microbial composition of the reagent. Notably, the NIBSC WC-Gut RR that used fastidious anaerobic bacteria that are common in the gut and often hard to lyse proved a much stronger challenge for DNA extraction kits than those based on common clinical bacteria that would rarely be found in a healthy gut. This emphasises the importance of reagents that are specific to the site of interest to prevent inflated measures of benchmarks that could occur when using bacteria that are easy to lyse and commonly found in databases. It also highlights the importance of the transparent validation of reagents through publication and peer review. A limitation of these reagents is that they do not account for inhibition and difficulties posed by the matrix or host DNA. However, we believe they do represent a good control for DNA extractions. If users cannot accurately get acceptable results in physicochemical characteristics and good levels of accuracy in the four-reporting measures of taxonomic composition using such WC-Gut RR, they would almost certainly fail to achieve accuracy in results when extracting DNA from clinical samples. In future work, we plan to establish minimum quality criteria for the four measures of taxonomic composition using an inter-collaborative study in 2022. This study will also be used to establish the reagent as the 1st WHO International Whole Cell Reference Reagent for DNA extractions of microbiome samples.

The NIBSC Whole Cell Reference Reagent will act as complementary to the NIBSC DNA Reference Reagent, which is now the 1st WHO International Reference Reagent for microbiome analysis by NGS and targets the library preparation, sequencing, and bioinformatics bias (Amos et al., 2020). Furthermore, this study indicates the need for site-specific reagents, so we are developing site-specific reference reagents for lung, nasopharynx, oral, skin, and vaginal microbiome. Besides this, the processes involved in the microbiome field, e.g. sampling, storage, DNA extraction, bioinformatics analysis, and the bias that each of them introduces, need to be tackled using process-specific reagents. Following the NIBSC DNA Reference Reagents and the NIBSC Whole Cell Reference Reagents, there is a need to produce standards that will eliminate the sampling and storage bias [17, 53, 54], in order to standardise all the steps involved in the process.

## Conclusions

Despite the clear successes of microbiome research and exciting applications that its results suggest, the field is suffering from limited reproducibility and comparability between studies [3–9]. This can lead to very large expensive but abstract studies being performed that are not comparable and, more worryingly, a situation where clinical trials are conducted with methodologies that are not fit for purpose. This situation is particularly acute considering the sheer number of early-stage therapeutics based on microbiome studies [55–57]. We developed the NIBSC Whole Cell Reference Reagent to be used as a reference point for the efficiency of the DNA extractions of microbiome samples and to facilitate benchmarking and validating protocols. We demonstrated that this reagent can reveal bias introduced during the DNA extraction process using a straightforward and easy-to-use four-measure reporting system. These reagents will undergo an inter-collaborative study in 2022 in an effort to establish minimum reporting criteria for use with these reagents.

## Supplementary Information

**Additional file 1: Supplementary Table 1-11.**

**Additional file 2: Supplementary Figure 1.** Microscopy images indicating that cells are intact with the DNA within the cells after fixation and lyophilisation. A) Confocal microscopy images of acetone fixed bacteria stained with Acridine Orange (AO), setting adjusted to allow observation of DNA, RNA, Differential Interference Contrast (DIC), and an Overlay of all three. Images presented are maximum intensity projections of confocal Z stacks, B) EM images of bacterial cells, black arrows indicate the presence of granular nucleoplasm with fibrillar whorls inside the cell membranes, indicating that DNA is preserved within the cells post fixation and lyophilisation.

**Additional file 3: Supplementary Figure 2.** Comparison of the taxonomic composition of samples before and after lyophilisation (pre-lyo and post-lyo respectively). Samples analysed are the Actual (ground truth), the NIBSC DNA Gut-Mix-RR and DNA extracted from the WC-Gut-RR using Kit1, Kit2, Kit3, Kit4, Kit5, Kit6, Kit7, Kit8 before and after lyophilisation. A) Relative abundance of (%) of each species in the samples analysed using Shotgun Sequencing and the MetaPlAn3 pipeline, B) Similarity (%) scores of the microbial composition before and after lyophilisation calculated using DNA extracted from the eight different kits, sequenced with Shotgun Sequencing and analysed using the MetaPlAn3 pipeline, C) Relative abundance of (%) of each genera in the samples analysed using 16S Amplicon Sequencing and the QIIME2 (Deblur) pipeline, B) Similarity (%) scores of the microbial composition before and after lyophilisation calculated using DNA extracted from the eight different kits, sequenced with 16S Amplicon Sequencing and analysed using the QIIME2 (Deblur) pipeline.

**Additional file 4: Supplementary Figure 3.** Comparison of DNA extraction kits and their extraction biases using Shotgun Sequencing and 4 different bioinformatics tools. A)Centrifuge. B)Kaiju. C)Kraken. D)Bracken. Samples analysed are the Actual (ground truth), the NIBSC DNA-Gut-Mix-RR and DNA extracted from the WC-Gut-RR using Kit1, Kit2, Kit3, Kit4, Kit5, Kit6, Kit7, Kit8. The barplots show the taxonomic composition and the relative abundance (%) of each taxon in each sample and the tables contain the scores of each sample for the reporting measures; Sensitivity, False-Positive Relative Abundance (FPRA), Similarity the Actual composition and Similarity to the NIBSC DNA-Gut-Mix-RR.

**Additional file 5: Supplementary Methods.**

## Data Availability

All sequencing data generated through this study is publicly available through the NCBI Sequence Read Archive upon publication (PRJNA855455). All NIBSC-generated reference reagents are available through request from the corresponding author. From 2022 onwards, they will be made available through the NIBSC website. https://www.nibsc.org/.

## Abbreviations

AO: Acridine orange
ASV: Amplicon sequence variant
CLSM: Confocal laser scanning microscope
CV: Coefficient of variation
DIN: DNA integrity number
DSMZ or DSM: Deutsche Sammlung von Mikroorganismen und Zellkulturen
EM: Electron microscopy
FMT: Faecal microbiota transplant
FPRA: False-positive relative abundance
IHMS: International Human Microbiome Standards
MBQC: Microbiome Quality Control project
MQC: Minimum quality criteria
NGS: Next-generation sequencing
NIBSC: National Institute for Biological Standards and Control
PBS: Phosphate-buffered saline
RR: Reference reagent
WC: Whole cell
WHO: World Health Organization
