# Supplementary material for: Developing whole cell standards for the microbiome field
Source: Microbiome. 2022 Aug 9;10:123. doi: 10.1186/s40168-022-01313-z (PMC9361656; doi:10.1186/s40168-022-01313-z)
Supplement: Supplementary file 2 — Additional file 1: Supplementary Table 1-11. [file 40168_2022_1313_MOESM1_ESM.pdf]

Supplementary data

Supplementary Table 1: Physiochemical measures of DNA extracted from the fixed and non-fixed E. coli cells based on averages of three replicates.

	Yield (ng)		pairwise t-test FDR adj. p-value	Integrity (DIN)		pairwise t-test FDR adj. p-value	Purity (260/280 ratio)		pairwise t-test FDR adj. p-value
	fixed	non-fixed		fixed	non-fixed		fixed	non-fixed	
Kit1	7120	6740	0.85	6.20	6.20	1	1.93	1.93	0.996
Kit2	7306.7	8113.3	0.72	5.63	5.67	0.91	1.90	1.93	0.996
Kit3	331.3	151.5	0.93	n/a	n/a	n/a	n/a	n/a	n/a
Kit4	2060	3393.3	0.53	5.90	6.00	0.75	2.09	2.01	0.923
Kit5	4593.3	2813.3	0.39	7.17	7.07	0.75	1.97	2.01	0.996
Kit6	9766.7	10420	0.77	7.63	7.67	0.91	1.91	1.90	0.996
Kit7	2713.3	3953.3	0.56	6.47	6.80	0.27	2.00	2.00	0.996
Kit8	12373.3	11773.3	0.78	5.97	5.87	0.75	1.92	1.91	0.996

Supplementary Table 2: Physiochemical measures of DNA extracted from the NIBSC WC-Gut-RR before (average of three replicates) and after lyophilisation (average of five replicates)

	Yield (ng)		pairwise t-test FDR adj. p-value	Integrity (DIN)		pairwise t-test FDR adj. p-value	Purity (260/280 ratio)		pairwise t-test FDR adj. p-value
	Pre-lyo	Post-lyo		Pre-lyo	Post-lyo		Pre-lyo	Post-lyo	
Kit1	653	840	0.63	4.67	4.12	0.23	1.87	1.98	0.91
Kit2	655	799	0.73	4.93	4.28	0.17	1.89	1.69	0.65
Kit3	84	125	0.92	1.20	1.45	n/a	1.33	2	0.03
Kit4	883	1240	0.39	4.53	4.24	0.55	2.32	1.81	0.11
Kit5	499	628	0.79	4.53	3.35	0.01	2.04	1.96	0.91
Kit6	2187	2462	0.16	5.47	5.32	0.77	1.99	1.98	0.98
Kit7	382	555	0.66	5.55	4.84	0.18	1.58	1.81	0.60
Kit8	1677	1555	0.77	4.03	4.34	0.54	1.89	1.95	0.93

Supplementary Table 3: Technical specifications of the DNA extraction kits used. Steps included in the manufacturer's manual for each kit are indicated using the symbol "+" and steps not included are indicated with "-".

	Kit	Company	Specific for faeces	Beating Beads	Heat incubation	Cold incubation	Spin column purification	Filter after elution
Kit1	QIAamp PowerFecal DNA kit	Qiagen	+	+	+	+	+	-
Kit2	DNAeasyPowerSoil DNA Isolation Kit	Qiagen	-	+	-	+	+	-
Kit3	QIAamp Fast DNA stool Mini Kit	Qiagen	+	-	+	-	+	-
Kit4	ZymoBIOMICS DNA miniprep kit	Zymo Research	-	+	-	-	+	+
Kit5	GenElute™ Stool DNA Isolation Kit	Sigma-Aldrich	+	+	-	-	+	-
Kit6	FastDNA™ SPIN Kit for Soil	MP Biomedicals	-	+	-	-	+	-
Kit7	PureLink™ Microbiome DNA Purification Kit	Thermo Fisher Scientific	+	+	+	-	+	-
Kit8	DNeasy® PowerSoil® Pro Kit	Qiagen	-	+	-	-	+	-

Supplementary Table 4: Physiochemical measures of DNA extracted from the NIBSC WC-Gut-RR after lyophilisation; average based on five replicates and the Coefficient of Variation (CV%) of DNA Yield, DNA Integrity, DNA Purity.

**Post-Lyophilisation**							
		**Yield (ng)**		**Integrity (DIN)**		**Purity (260/280 ratio)**	
**Kit**		**Average**	**CV%**	**Average**	**CV%**	**Average**	**CV%**
QIAamp PowerFecal DNA kit	**Kit1**	840	11	4.12	1	1.98	7
Dneasy PowerSoil DNA Isolation Kit	**Kit2**	799	8	4.28	4	1.69	11
QIAamp Fast DNA stool Mini Kit	**Kit3**	125	13	1.45	15	2	31
ZymoBIOMICS DNA miniprep kit	**Kit4**	1240	9	4.24	4	1.81	3
GenElute™ Stool DNA Isolation Kit	**Kit5**	628	8	3.35	45	1.96	5
FastDNA™ SPIN Kit for Soil	**Kit6**	2462	10	5.32	1	1.98	2
PureLink Microbiome DNA Purification Kit	**Kit7**	555	17	4.84	15	1.81	14
DNeasy PowerSoil Pro Kit	**Kit8**	1555	3	4.34	2	1.95	2

Supplementary Table 5: FDR adjusted p-value calculated using the Kruskal-Wallis test, comparing the relative abundance of each strain in DNA-RR with those found in the WC-RR and of which DNA

extracted from each kit, FDR adjusted p-value > 0.05 is shown in bold. The data was analysed using Shotgun Sequencing and the MetaPlAn3 bioinformatics tool.

	Shotgun Sequencing / MetaPlAn3							
Taxonomy	Kit1	Kit2	Kit3	Kit4	Kit5	Kit6	Kit7	Kit8
Akkermansia muciniphila	**0.028**	**0.030**	**0.027**	**0.033**	**0.027**	**0.027**	**0.030**	**0.031**
Alistipes finegoldii	**0.028**	**0.030**	**0.027**	**0.033**	**0.027**	**0.027**	**0.030**	**0.031**
Anaerostipes hadrus	**0.028**	**0.030**	**0.027**	**0.033**	**0.027**	**0.027**	**0.030**	**0.031**
Bacteroides thetaiotaomicron	**0.028**	**0.030**	**0.027**	**0.033**	**0.027**	**0.027**	**0.030**	**0.031**
Bacteroides uniformis	**0.028**	**0.030**	**0.027**	**0.033**	**0.027**	**0.027**	**0.030**	**0.031**
Bifidobacterium longum	**0.028**	**0.030**	**0.027**	0.210	**0.027**	**0.027**	**0.030**	**0.031**
Blautia wexlerae	**0.028**	**0.030**	**0.027**	**0.033**	**0.027**	**0.027**	**0.030**	**0.031**
Clostridium butyricum	**0.028**	**0.030**	**0.027**	**0.033**	**0.027**	**0.027**	**0.030**	**0.031**
Collinsella aerofaciens	**0.028**	**0.030**	**0.027**	**0.033**	**0.027**	**0.027**	**0.030**	**0.031**
Escherichia coli	**0.028**	**0.030**	**0.027**	**0.065**	**0.027**	**0.027**	**0.030**	0.687
Eubacterium hallii	**0.028**	**0.030**	**0.027**	**0.033**	**0.027**	**0.027**	0.189	**0.031**
Faecalibacterium prausnitzii	**0.028**	**0.030**	**0.027**	0.479	**0.027**	**0.027**	**0.030**	0.118
Lactobacillus gasseri	**0.028**	**0.030**	**0.027**	**0.033**	**0.027**	**0.027**	**0.030**	**0.031**
Parabacteroides distasonis	**0.028**	**0.030**	**0.027**	**0.033**	**0.027**	**0.027**	**0.030**	**0.031**
Prevotella copri	**0.028**	**0.030**	**0.027**	**0.033**	**0.027**	**0.027**	**0.030**	**0.031**
Prevotella melaninogenica	**0.028**	**0.030**	**0.027**	**0.033**	**0.027**	**0.027**	**0.030**	**0.031**
Roseburia hominis	**0.028**	**0.030**	**0.027**	**0.033**	**0.027**	**0.027**	**0.030**	**0.031**
Roseburia intestinalis	**0.028**	0.106	**0.027**	**0.033**	**0.027**	**0.027**	**0.030**	**0.031**

Supplementary Table 6: P-value calculated via PERMANOVA, comparing the b-diversity (Bray-Curtis matrix) of the samples. The data was generated using Shotgun Sequencing and the MetaPlAn3 bioinformatics tool.

	PERMANOVA, p-value							
	DNA-Gut-Mix-RR	Kit1	Kit2	Kit3	Kit4	Kit5	Kit6	Kit7
Kit1	0.023							
Kit2	0.023	0.020						
Kit3	0.026	0.020	0.020					
Kit4	0.024	0.022	0.020	0.020				
Kit5	0.020	0.020	0.020	0.020	0.020			
Kit6	0.026	0.020	0.020	0.020	0.020	0.020		
Kit7	0.022	0.020	0.020	0.020	0.020	0.020	0.020	
Kit8	0.020	0.020	0.020	0.020	0.020	0.020	0.020	0.020

Supplementary Table 7: FDR adjusted p-value calculated using Tukey HSD following ANOVA, comparing the relative abundance of Gram-negative bacteria, using DNA extracted from the different DNA extraction kits, analysed using Shotgun Sequencing and the MetaPlAn3 bioinformatics tool.

Tukey HSD following ANOVA, FDR adjusted p-value								
	DNA-Gut-Mix-RR	Kit1	Kit2	Kit3	Kit4	Kit5	Kit6	Kit7
Kit1	7.09E-10							
Kit2	5.91E-11	0.96						
Kit3	0	0	0					
Kit4	1.64E-13	0	0	0				
Kit5	0	0	0	0.13	0			
Kit6	0	0	0	0	0	0		
Kit7	2.79E-05	0	0	0	3.07E-08	0	0	
Kit8	1.38E-10	0	0	0	0.056	0	0	0.00038

Supplementary Table 8: FDR adjusted p-value calculated using the Kruskal-Wallis test, comparing the relative abundance of each strain in NIBSC DNA-Gut-Mix-RR with those found in the WC-RR and of which DNA extracted from each kit, FDR adjusted p-value > 0.05 is shown in bold. The data was analysed using 16S Amplicon Sequencing and the QIIME2 (Deblur) pipeline.

16S Amplicon Sequencing / QIIME2 – Deblur								
Taxonomy	Kit1	Kit2	Kit3	Kit4	Kit5	Kit6	Kit7	Kit8
Akkermansia	**0.031**	**0.029**	**0.027**	0.456	**0.029**	**0.029**	0.205	0.486
Alistipes	**0.031**	**0.029**	**0.027**	**0.034**	**0.029**	**0.029**	**0.031**	**0.031**
Anaerostipes	**0.031**	**0.029**	**0.027**	**0.034**	0.297	0.192	**0.031**	**0.031**
Bacteroides	**0.031**	**0.029**	**0.027**	**0.034**	**0.029**	**0.029**	**0.031**	**0.031**
Bifidobacterium	**0.031**	**0.029**	**0.027**	0.456	**0.029**	**0.029**	0.486	0.655
Blautia	**0.031**	**0.029**	**0.027**	**0.034**	**0.029**	**0.029**	**0.031**	**0.031**
Clostridium	**0.031**	**0.029**	**0.027**	**0.034**	**0.029**	**0.029**	**0.031**	**0.031**
Collinsella	**0.031**	**0.029**	**0.027**	**0.034**	**0.029**	**0.029**	**0.031**	**0.031**
Escherichia	**0.031**	**0.029**	**0.027**	**0.034**	**0.029**	**0.029**	0.881	**0.031**
Eubacterium	**0.031**	**0.029**	**0.027**	**0.034**	**0.029**	**0.029**	**0.031**	**0.031**
Faecalibacterium	**0.031**	**0.029**	**0.027**	0.205	**0.029**	**0.029**	**0.031**	**0.031**
Lactobacillus	0.316	**0.029**	**0.027**	0.124	**0.029**	**0.029**	**0.031**	0.339
Parabacteroides	**0.031**	**0.029**	**0.027**	**0.034**	**0.029**	**0.029**	**0.031**	**0.031**
Prevotella	**0.031**	**0.029**	0.053	**0.034**	**0.029**	**0.029**	**0.031**	**0.031**
Roseburia	0.881	0.655	**0.027**	**0.034**	**0.029**	**0.029**	**0.031**	**0.031**
Ruminococcus	0.316	0.316	**0.027**	**0.034**	0.192	0.655	**0.031**	**0.031**

Supplementary Table 9: P-value calculated via PERMANOVA, comparing the b-diversity (Bray-Curtis matrix) of the samples. The data was generated using 16S Amplicon Sequencing and the QIIME2 (Deblur) pipeline.

PERMANOVA, p-value								
	DNA-Gut-Mix-RR	Kit1	Kit2	Kit3	Kit4	Kit5	Kit6	Kit7
Kit1	0.025							
Kit2	0.031	0.105						
Kit3	0.034	0.022	0.025					
Kit4	0.031	0.022	0.022	0.022				
Kit5	0.030	0.022	0.022	0.022	0.022			
Kit6	0.025	0.022	0.024	0.022	0.022	0.022		
Kit7	0.03	0.022	0.022	0.022	0.022	0.022	0.022	
Kit8	0.022	0.022	0.022	0.022	0.545	0.022	0.022	0.022

Supplementary Table 10: FDR adjusted p-value calculated using Tukey HSD following ANOVA, comparing the relative abundance of Gram-negative bacteria, using DNA extracted from the different DNA extraction kits, analysed using Shotgun Sequencing and the MetaPlAn3 bioinformatics tool.

Tukey HSD following ANOVA, FDR adjusted p-value								
	DNA-Gut-Mix-RR	Kit1	Kit2	Kit3	Kit4	Kit5	Kit6	Kit7
Kit1	0.041							
Kit2	0.015	0.9999						
Kit3	0	0	0					
Kit4	8.15E-05	1.4E-10	4.45E-11	0				
Kit5	0	0	0	0.023	0			
Kit6	0	2.67E-13	8.02E-13	0.017	0	5.2E-07		
Kit7	1	0.0053	0.0014	0	1.38E-05	0	0	
Kit8	0.00074	1.32E-09	3.96E-10	0	0.993	0	0	0.00018

Supplementary Table 11: FDR adjusted p-value calculated using Tukey HSD following ANOVA, comparing the relative abundance of Gram-positive bacteria, using DNA extracted from the different DNA extraction kits, using five different mock communities; NIBSC WC-RR, ZYMO, ATCC-MSA 2002,

ATCC-MSA 2003, ATCC-MSA 2006. The data was generated using Shotgun Sequencing and the MetaPlAn3 bioinformatics tool.

**NIBSC WC-RR**			
	Kit2	Kit6	Kit7
DNA-Gut-Mix-RR	1	8.03E-05	0.000226
Kit2		9.41E-05	0.000193
Kit6			3.6E-11

**ZYMO**		
	Kit6	Kit7
Kit2	0.073592	4.9E-13
Kit6		1.01E-13

**ATCC-MSA 2002**		
	Kit6	Kit7
Kit2	0.38887	3.01E-13
Kit6		1.02E-13

**ATCC-MSA 2003**		
	Kit6	Kit7
Kit2	0.977396	1.19E-06
Kit6		7.19E-05

**ATCC-MSA 2006**		
	Kit6	Kit7
Kit2	4.63E-05	1.04E-13
Kit6		1.35E-09

Supplementary Table 12: Composition of commercial reagents used in this study.

ATCC-MSA 2002		
Species	Composition (%)	Gram status
Acinetobacter baumannii	5	-
Actinomyces odontolyticus	5	+
Bacillus cereus	5	+
Bacteroides vulgatus	5	-
Bifidobacterium adolescentis	5	+
Clostridium beijerinckii	5	+
Cutibacterium acnes	5	+
Deinococcus radiodurans	5	+
Enterococcus faecalis	5	+
Escherichia coli	5	-
Helicobacter pylori	5	-
Lactobacillus gasseri	5	+
Neisseria meningitidis	5	-
Porphyromonas gingivalis	5	-
Pseudomonas aeruginosa	5	-
Rhodobacter sphaeroides	5	-
Staphylococcus aureus	5	+
Staphylococcus epidermidis	5	+
Streptococcus agalactiae	5	+
Streptococcus mutans	5	+
ATCC-MSA 2003		
Species	Composition (%)	Gram status
Bacillus cereus	10	+
Bifidobacterium adolescentis	10	+
Clostridium beijerinckii	10	+
Deinococcus radiodurans	10	+
Enterococcus faecalis	10	+
Escherichia coli	10	-
Lactobacillus gasseri	10	+
Rhodobacter sphaeroides	10	-
Staphylococcus epidermidis	10	+
Streptococcus mutans	10	+
ATCC-MSA 2006		
Species	Composition (%)	Gram status
Bacteroides fragilis	8.3	-
Bacteroides vulgatus	8.3	-
Bifidobacterium adolescentis	8.3	+
Clostridioides difficile	8.3	+
Enterococcus faecalis	8.3	+
Escherichia coli	8.3	-
Fusobacterium nucleatum	8.3	-
Helicobacter pylori	8.3	-
Lactobacillus plantarum	8.3	+
Salmonella enterica	8.3	-
Yersinia enterocolitica	8.3	-
Enterobacter cloacae	8.3	-
ZYMO		
Species	Composition (%)	Gram status
Bacillus intestinalis	12	+
Enterococcus faecalis	12	+
Escherichia coli	12	-
Lactobacillus fermentum	12	+
Listeria monocytogenes	12	+
Pseudomonas aeruginosa	12	-
Salmonella enterica	12	-
Staphylococcus aureus	12	+
