# Supplementary figures and images for: Developing whole cell standards for the microbiome field
Source: Microbiome. 2022 Aug 9;10:123. doi: 10.1186/s40168-022-01313-z (PMC9361656; doi:10.1186/s40168-022-01313-z)

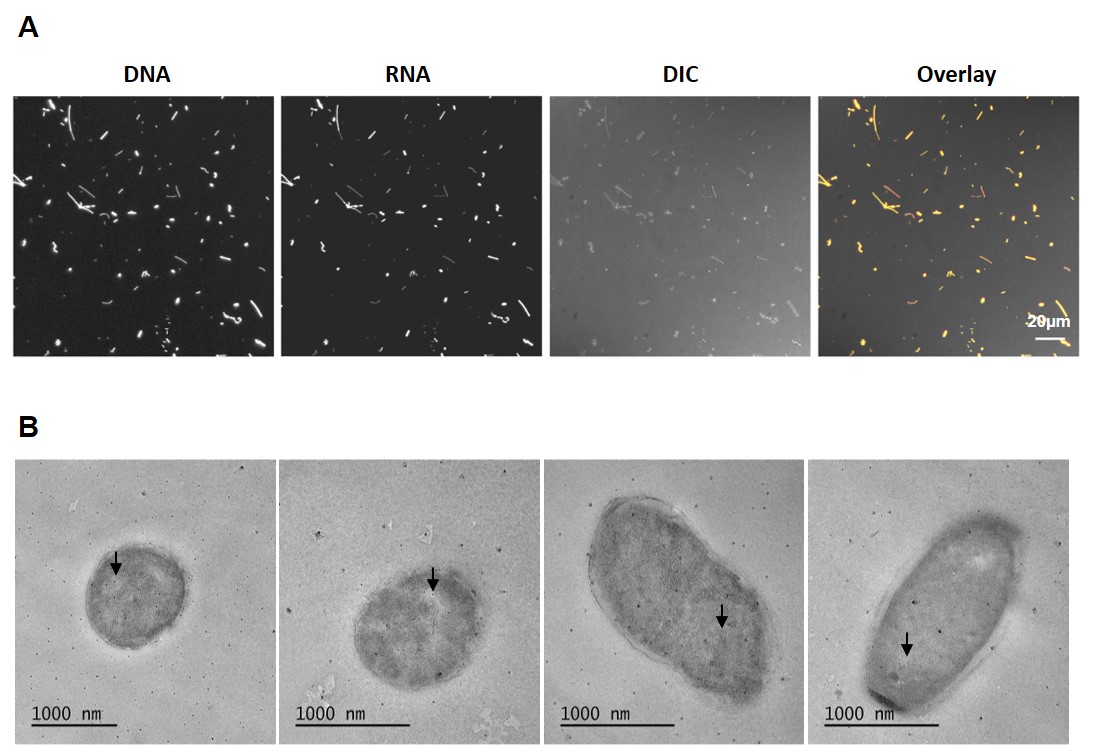

Supplement: Supplementary file 3 — Additional file 2: Supplementary Figure 1. Microscopy images indicating that cells are intact with the DNA within the cells after fixation and lyophilisation. A) Confocal microscopy images of acetone fixed bacteria stained with Acridine Orange (AO), setting adjusted to allow observation of DNA, RNA, Differential Interference Contrast (DIC), and an Overlay of all three. Images presented are maximum intensity projections of confocal Z stacks, B) EM images of bacterial cells, black arrows indicate the presence of granular nucleoplasm with fibrillar whorls inside the cell membranes, indicating that DNA is preserved within the cells post fixation and lyophilisation. [file 40168_2022_1313_MOESM2_ESM.jpg]
