# Supplementary material for: Developing whole cell standards for the microbiome field
Source: Microbiome. 2022 Aug 9;10:123. doi: 10.1186/s40168-022-01313-z (PMC9361656; doi:10.1186/s40168-022-01313-z)
Supplement: Supplementary file 4 — Additional file 3: Supplementary Figure 2. Comparison of the taxonomic composition of samples before and after lyophilisation (pre-lyo and post-lyo respectively). Samples analysed are the Actual (ground truth), the NIBSC DNA Gut-Mix-RR and DNA extracted from the WC-Gut-RR using Kit1, Kit2, Kit3, Kit4, Kit5, Kit6, Kit7, Kit8 before and after lyophilisation. A) Relative abundance of (%) of each species in the samples analysed using Shotgun Sequencing and the MetaPlAn3 pipeline, B) Similarity (%) scores of the microbial composition before and after lyophilisation calculated using DNA extracted from the eight different kits, sequenced with Shotgun Sequencing and analysed using the MetaPlAn3 pipeline, C) Relative abundance of (%) of each genera in the samples analysed using 16S Amplicon Sequencing and the QIIME2 (Deblur) pipeline, B) Similarity (%) scores of the microbial composition before and after lyophilisation calculated using DNA extracted from the eight different kits, sequenced with 16S Amplicon Sequencing and analysed using the QIIME2 (Deblur) pipeline. [file 40168_2022_1313_MOESM3_ESM.pdf]

A

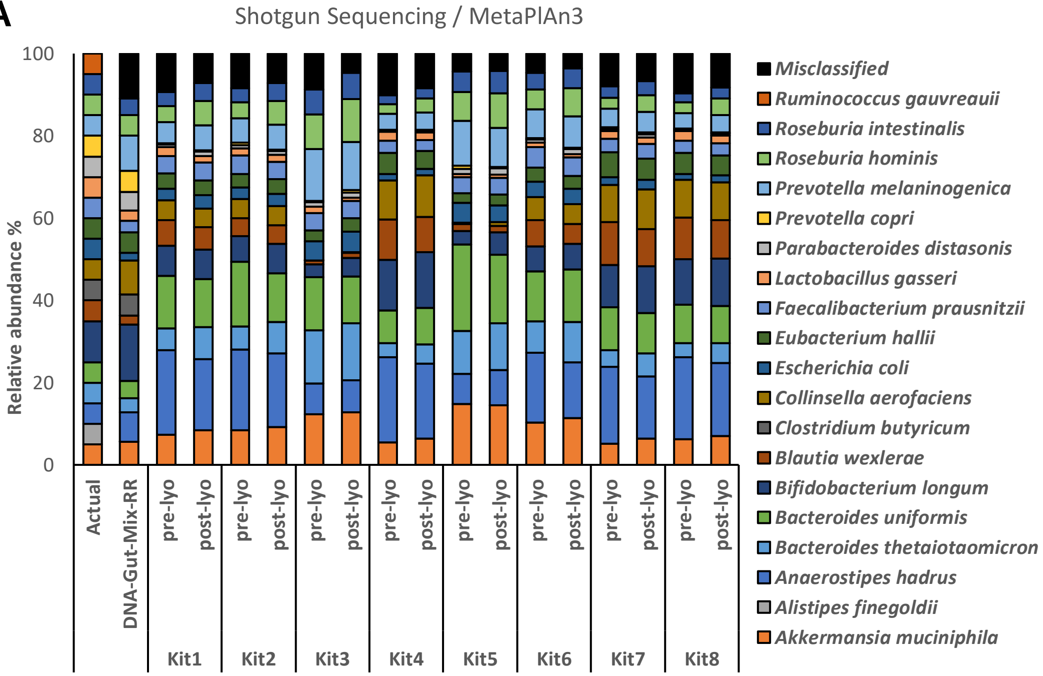

B

MetaPlAn3	Similarity %
Kit1	91.6
Kit2	92.4
Kit3	92.8
Kit4	93.4
Kit5	92.5
Kit6	92.1
Kit7	92.1
Kit8	94.3

C

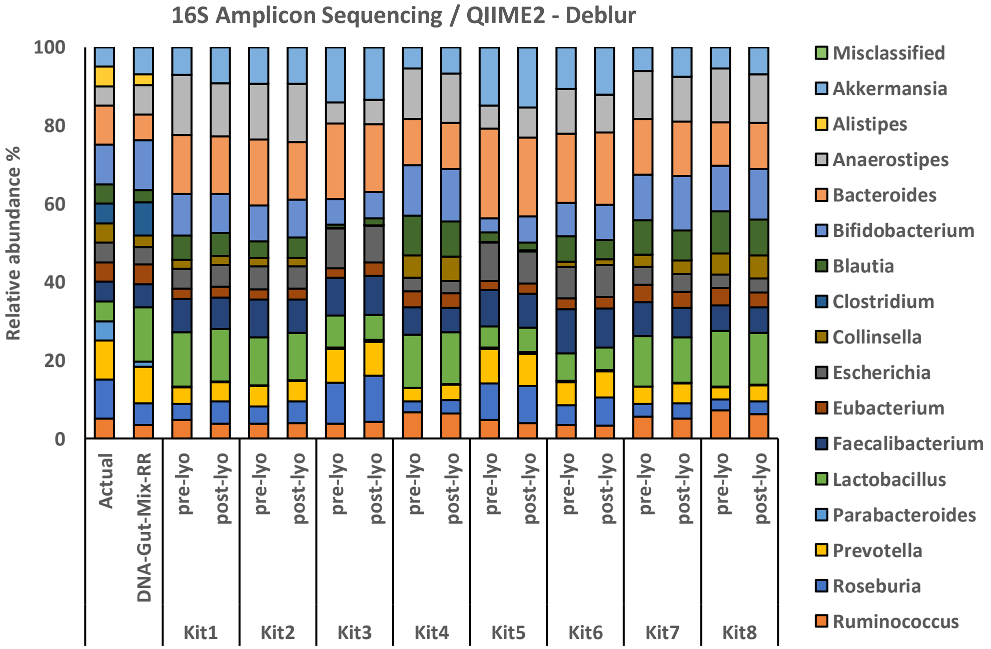

D

16S	Similarity %
Kit1	94.7
Kit2	96.3
Kit3	94.8
Kit4	96.7
Kit5	93.1
Kit6	94.0
Kit7	94.4
Kit8	94.5
