# Supplementary material for: Developing whole cell standards for the microbiome field
Source: Microbiome. 2022 Aug 9;10:123. doi: 10.1186/s40168-022-01313-z (PMC9361656; doi:10.1186/s40168-022-01313-z)
Supplement: Supplementary file 5 — Additional file 4: Supplementary Figure 3. Comparison of DNA extraction kits and their extraction biases using Shotgun Sequencing and 4 different bioinformatics tools. A)Centrifuge. B)Kaiju. C)Kraken. D)Bracken. Samples analysed are the Actual (ground truth), the NIBSC DNA-Gut-Mix-RR and DNA extracted from the WC-Gut-RR using Kit1, Kit2, Kit3, Kit4, Kit5, Kit6, Kit7, Kit8. The barplots show the taxonomic composition and the relative abundance (%) of each taxon in each sample and the tables contain the scores of each sample for the reporting measures; Sensitivity, False-Positive Relative Abundance (FPRA), Similarity the Actual composition and Similarity to the NIBSC DNA-Gut-Mix-RR. [file 40168_2022_1313_MOESM4_ESM.pdf]

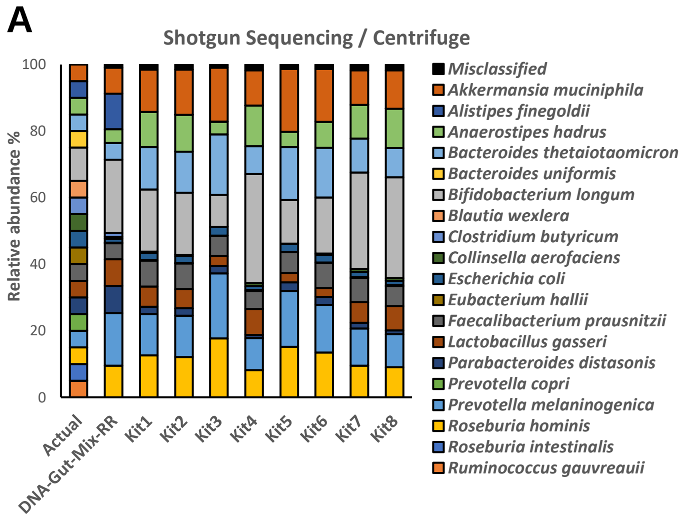

	Sensitivity	FPRA	Diversity	Similarity to Actual	Similarity to DNA-Gut-Mix-RR
Actual	100	0.0	19	100	60
DNA-Gut-Mix-RR	79	0.9	38	60	100
Kit1	74	1.6	42	50	73
Kit2	74	1.5	42	50	73
Kit3	74	0.9	36	46	63
Kit4	74	1.7	45	48	73
Kit5	74	1.3	37	48	68
Kit6	74	1.3	40	48	70
Kit7	74	1.7	45	50	75
Kit8	74	1.7	45	49	74

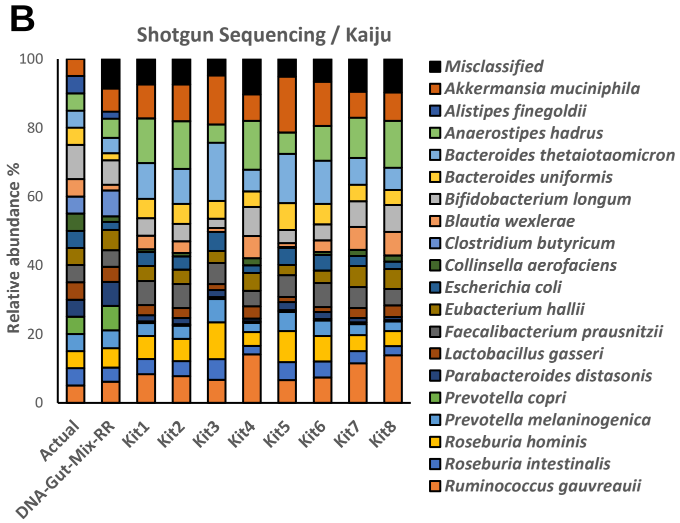

	Sensitivity	FPRA	Diversity	Similarity to Actual	Similarity to DNA-Gut-Mix-RR
Actual	100	0.0	19	100	71
DNA-Gut-Mix-RR	100	8.6	207	71	100
Kit1	95	7.4	196	65	69
Kit2	100	7.4	200	64	69
Kit3	89	4.8	167	61	62
Kit4	89	10.3	202	61	69
Kit5	95	5.1	169	62	65
Kit6	95	6.7	184	64	67
Kit7	89	9.5	197	65	73
Kit8	89	9.7	198	62	70

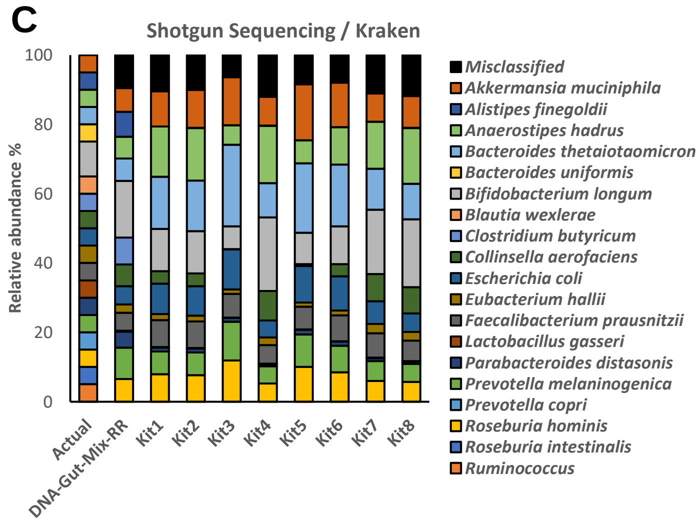

	Sensitivity	FPRA	Diversity	Similarity to Actual	Similarity to DNA-Gut-Mix-RR
Actual	100	0.0	19	100	68
DNA-Gut-Mix-RR	74	9.5	84	68	100
Kit1	68	10.5	82	56	69
Kit2	68	10.1	82	56	69
Kit3	68	6.5	68	47	59
Kit4	68	12.0	86	55	75
Kit5	68	8.5	70	50	63
Kit6	68	8.0	79	55	66
Kit7	68	11.1	84	59	77
Kit8	68	11.9	84	56	77

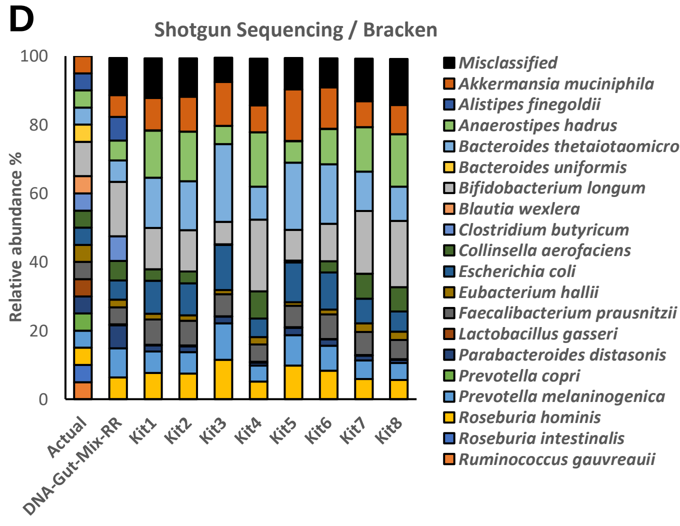

	Sensitivity	FPRA	Diversity	Similarity to Actual	Similarity to DNA-Gut-Mix-RR
Actual	100	0.0	19	100	66
DNA-Gut-Mix-RR	74	10.9	94	66	100
Kit1	74	11.6	91	52	69
Kit2	68	11.3	90	52	69
Kit3	68	7.1	72	45	59
Kit4	68	13.7	95	53	73
Kit5	68	9.1	78	48	63
Kit6	68	8.6	87	52	66
Kit7	74	12.4	95	54	74
Kit8	68	13.5	93	54	74
