# Supplementary material for: Developing whole cell standards for the microbiome field
Source: Microbiome. 2022 Aug 9;10:123. doi: 10.1186/s40168-022-01313-z (PMC9361656; doi:10.1186/s40168-022-01313-z)
Supplement: Supplementary file 6 — Additional file 5: Supplementary Methods. [file 40168_2022_1313_MOESM5_ESM.pdf]

Supplementary methods

Analysis of Shotgun Sequencing

For analysis of shotgun metagenomic sequencing data we used the following commands:

1) Used the tool BBDuk to quality control the sequences.

```
bbduk.sh -Xmx20g in1=forward.fastq in2=reverse.fastq out1=cleanforward.fastq \
out2=cleanreverse.fastq ref=/filepath/bbmap/resources/adapters.fa ktrim=r k=21
mink=10 \
hdist=2 qtrim=r trimq=25 minlen=100 t=7
```

2) We did not subsample the files, in order to take into account any bias introduced by the DNA extraction kits. The DNA extracted from the WC-Gut-RR using different DNA extraction kits was sequence with an average of 4.8 Million reads per sample (Table SM1A) whereas the DNA extracted from different mock communities and different DNA extraction kits had variable sequence coverage (Table SM1B).

Table SM1: Average million reads yielded for each sample using Shotgun sequencing.

A

Samples	Average reads (Million)
DNA-Gut-Mix-RR	4.9 ± 0.09
Kit1	4.3 ± 0.33
Kit2	4.4 ± 0.43
Kit3	5.4 ± 1.97
Kit4	5.6 ± 0.9
Kit5	4.3 ± 0.63
Kit6	4.8 ± 0.46
Kit7	4.9 ± 0.31
Kit8	5.5 ± 1.41

B

Samples		Average reads (Million)
DNA-Gut-Mix-RR		1.2 ± 0.32
NIBSC	Kit2	1.6 ± 0.02
	Kit6	1.5 ± 0.31
	Kit7	1.3 ± 0.39
Zymo	Kit2	1.0 ± 0.17
	Kit6	0.9 ± 0.34
	Kit7	1.5 ± 0.55
ATCC-2002	Kit2	1.4 ± 0.09
	Kit6	0.9 ± 0.05
	Kit7	1.4 ± 0.11
ATCC-2003	Kit2	1.3 ± 0.18
	Kit6	1.5 ± 0.17
	Kit7	1.5 ± 0.13
ATCC-2006	Kit2	1.1 ± 0.29
	Kit6	0.8 ± 0.11
	Kit7	1.1 ± 0.05

3) Meta-analysis of the combined sequencing files using Kaiju

```
kaiju -t $kaijudb/nodes.dmp -f $kaijudb/kaiju_db.fmi -i $i\_clean.fastq -j
$i\_clean.fastq -o $filepath/${i}_out -z 7 -a mem

kaiju2table -t $kaijudb/nodes.dmp -n $kaijudb/names.dmp -r species -m 0.005 -u -o
$filepath/${i}_species.txt $filepath/*_out.txt
```

4) Combined the clean files:

```
for f in *_clean_R1_001.fastq
```

```
do
prefix1=${f/R1_001.fastq/}
cat $f ${prefix1}R2_001.fastq > ${prefix1}_combined.fastq
done
```

5) Meta-analysis of the combined sequencing files using MetaPlAn3

```
metaphlan $filepath/cleancombined.fastq --input_type fastq \
--no_map --nproc 8 --index mpa_v30_CHOCOPhlAn_201901 > $filepath/profile.txt

merge_metaphlan_tables.py \
$filepath/profile.txt > $filepath/merged_table.txt
```

6) Meta-analysis of the combined sequencing files using Centrifuge

```
for f in *combined.fastq
do
centrifuge -x $filepath/centrifuge/indices/p_compressed  -U ${f} -p 7 -S
$filepath/${f}_report.txt --report-file
$filepath/Centrifuge/${f}_centrifuge_report.txt
done
```

7) To perform analysis using Kraken and Bracken we used paired end clean sequencing files.

```
forward=$filepath/cleanforward.fastq
reverse=$filepath/cleanreverse.fastq

kraken --threads 4 --paired --preload --db /raid/kraken/standard_kraken_db/ \
--classified-out $filepath/kraken.classified --unclassified-out
$filepath/kraken.unclassified \
--out $filepath/kraken.output $forward $reverse

kraken-mpa-report --db /raid/kraken/standard_kraken_db/ \
$filepath/kraken.output > $filepath/kraken.mpa.report

kraken-report --db /raid/kraken/standard_kraken_db/ \
$filepath/kraken.output > $filepath/kraken.report
```

8) Using the kraken.report produced in the previous step we run Bracken:

```
bracken -d /raid/kraken/standard_kraken_db/ \
-i $filepath/kraken.report -o $filepath/bracken.report -r 140 -t 5
```

The output files from steps 3, 5, 6, 7 and 8 were used for analysis in excel and/or R were used as described in the Methods of the main manuscript.

Analysis of 16S rRNA sequencing

To analyse 16S rRNA sequencing data please use the QIIME2 platform https://qiime2.org/, using VSEARCH for paired end merging and Deblur for denoising.

1) Import the data into QIIME2 creating a .qza QIIME2 artifact:

```
qiime tools import \
 --type 'SampleData[PairedEndSequencesWithQuality]' \
 --input-path $filepath \
```

```
 --input-format CasavaOneEightSingleLanePerSampleDirFmt \
 --output-path $filepath/demux.qza
```

2) Primers and adaptors are removed using the q2-cutadapt plugin:

```
qiime cutadapt trim-paired \
 --i-demultiplexed-sequences $filepath/demux.qza \
 --p-cores 4 \
 --p-adapter-f GTGYCAGCMGCCGCGGTAA...ATTAGAWACCCBNGTAGTCC \
 --p-adapter-r GGACTACNVGGGTWTCTAAT...TTACCGCGGCKGCTGRCAC \
 --output-dir $filepath/trimmed
```

3) To analyse the data through the deblur route, first join the trimmed sequences using VSEARCH through the q2-vsearch plugin:

```
qiime vsearch join-pairs \
 --i-demultiplexed-seqs $filepath/trimmed/trimmed_sequences.qza \
 --output-dir $filepath/deblur
```

4) Perform an additional quality filtering using the q2-quality-filter plugin.

```
qiime quality-filter q-score-joined \
 --i-demux $filepath/deblur/joined_sequences.qza \
 --o-filtered-sequences $filepath/deblur/QC_demux_trimmed-joined.qza \
 --o-filter-stats $filepath/deblur/qc_demux_stats.qza
```

5) Denoise the quality filtered files using the q2-deblur plugin.

```
qiime deblur denoise-16S \
  --i-demultiplexed-seqs $filepath/deblur/QC_demux_trimmed-joined.qza \
  --p-trim-length 250 \
  --o-representative-sequences $filepath/deblur/rep-seqs-deblur.qza \
  --o-table $filepath/deblur/table-deblur.qza \
  --p-jobs-to-start 4 \
  --p-sample-stats \
  --o-stats $filepath/deblur/deblur-stats.qza
```

6) To classify features, we used the q2-feature-classifier plugin using the classify sklearn method. Use a pre-trained classifier which was trained on the Silva 132 release with 99% OTUs from the 515F/806R, sourced from QIIME2 data resources

```
qiime feature-classifier classify-sklearn \
  --i-classifier $filepath/silva-132-99-515-806-nb-classifier.qza \
  --i-reads $filepath/deblur/rep-seqs-deblur.qza \
  --o-classification $filepath/deblur/taxonomy.qza \
  --p-n-jobs -1
```

7) Filter the feature table was produced by deblur to ensure we only included features present in all replicates:

```
qiime feature-table filter-features \
  --i-table $filepath/deblur/table-deblur.qza \
  --p-min-samples 5 \
  --o-filtered-table $filepath/deblur/sample-contingency-filtered-table.qza
```

8) To filter out all features which do not appear at an abundance of 0.005 % in the final feature table, visualise the contingency filtered feature table to calculate 0.005 % of the total read abundance and use this as a --p-min-frequency in the q2-feature-table plugin (filter-features).

```
qiime feature-table summarize \
 --i-table $filepath/deblur/sample-contingency-filtered-table.qza \
 --o-visualization $filepath/deblur/sample-contingency-filtered-table.qzv

qiime tools view $filepath/deblur/sample-contingency-filtered-table.qzv

qiime feature-table filter-features \
  --i-table $filepath/deblur/sample-contingency-filtered-table.qza \
  --p-min-frequency [input number here] \
  --o-filtered-table $filepath/deblur/final-filtered-table.qza
```

9) We performed alpha and beta diversity analysis using the qiime phylogeny and qiime diversity plugin, by subsampling at 40000 reads. The core metrics folder produced contains rarefied table to be used in the next step:

```
qiime phylogeny align-to-tree-mafft-fasttree \
  --i-sequences $filepath/deblur/rep-seqs-deblur.qza \
  --o-alignment $filepath/aligned-rep-seqs.qza \
  --o-masked-alignment $filepath/masked-aligned-rep-seqs.qza \
  --o-tree $filepath/unrooted-tree.qza \
  --o-rooted-tree $filepath/rooted-tree.qza


qiime diversity core-metrics-phylogenetic \
  --i-phylogeny $filepath/rooted-tree.qza \
  --i-table $filepath/deblur/final-filtered-table.qza \
  --p-sampling-depth 40000 \
  --m-metadata-file $filepath/metadata.txt \
  --output-dir $filepath/core-metrics
```

10) In order to generate taxa bar plots and visualize them we used the q2-taxa plugin with the rarefied table produced in the previous step:

```
qiime taxa barplot \
 --i-table $filepath/core-metrics/rarefied-table.qza \
 --i-taxonomy $filepath/deblur/taxonomy.qza \
 --o-visualization $filepath/deblur/barplots.qzv \
 --m-metadata-file $filepath/metadata.txt

qiime tools view $filepath/deblur/barplots.qzv
```

The CSV files from the barplots generated in step 10 were used as genera abundance tables. Genera abundance profiles were used as described in the Methods of the main manuscript.
